# Effects of parasitic freshwater mussels on their host fishes: a review

**DOI:** 10.1017/S0031182022001226

**Published:** 2022-12

**Authors:** Sebastian L. Rock, Johan Watz, P. Anders Nilsson, Martin Österling

**Affiliations:** 1 Biology, Karlstad University, 65188 Karlstad, Sweden; 2 Department of Biology – Aquatic Ecology, Lund University, 223 62 Lund, Sweden

**Keywords:** Conservation, freshwater mussel, glochidia, host effects, Unionida

## Abstract

Freshwater mussels in the order Unionida are highly adapted to parasitize fish for the primary purpose of dispersal. The parasitic larval stage affixes itself to the gills or fins of the host where it becomes encysted in the tissue, eventually excysting to develop into a free-living adult. Research on the parasitic interactions between unionids and their host fishes has garnered attention recently due to the increase in worldwide preservation efforts surrounding this highly endangered and ecologically significant order. With the exception of heavy infestation events, these mussels cause minor effects to their hosts, typically only observable effect in combination with other stressors. Moreover, the range of effect intensities on the host varies greatly with the species involved in the interaction, an effect that may arise from different evolutionary strategies between long- and short-infesting mussels; a distinction not typically made in conservation practices. Lower growth and reduced osmotic potential in infested hosts are commonly observed and correlated with infestation load. These effects are typically also associated with increases in metabolic rate and behaviour indicative of stress. Host fish seem to compensate for this through a combination of rapid wound healing in the parasitized areas and higher ventilation rates. The findings are heavily biased towards *Margaritifera margaritifera*, a unique mussel not well suited for cross-species generalizations. Furthermore, the small body of molecular and genetic studies should be expanded as many conclusions are drawn from studies on the ultimate effects of glochidiosis rather than proximate studies on the underlying mechanisms.

## Graphical abstract

**Figure:**
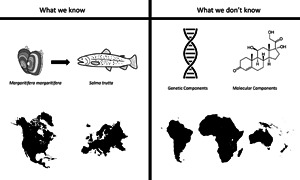

## Introduction

The relationships between parasites and their hosts are among the most complex and specialized forms of organism interactions on the planet. Interest in these interactions has grown rapidly since the inclusion of parasitology to the field of evolutionary ecology, as parasites have been shown to play a key role in regulating population size (Scott and Dobson, 1989; Tompkins *et al*., 2002), behaviour (Wesołowska and Wesołowski, 2014) and the general ecology of hosts (Poulin and FitzGerald, 1989), as well as shaping evolution (Rook, 2007; Parratt and Laine, 2016). For example, parasites have even been proposed as a driving factor behind the evolution of sexual reproduction (Hamilton *et al*., 1990). Perhaps the most widely accepted definition of the term parasite is ‘an organism that lives in or on another organism, the host, and causes it some harm by exploiting it through a structurally adapted way of life’ (Poulin, 2007). This definition can be applied to plants, fungi, animals, viruses, bacteria and, some argue, even to specific DNA strands (Combes*,* 2000, 2001; Poulin, 2007). The term parasite has also been described in an ecological sense as ‘predators that eat prey units of less than one’ (Wilson, 2014).

Given the vast diversity of parasitic organisms, the literature typically categorizes them according to the strategies that they have evolved. Among the factors taken into account when describing a parasite are the location on the host which the parasite infests, the number of hosts the parasite requires to complete its life cycle and the proportion of the life cycle spent as a parasite (Box 1). Parasites have been generally regarded as negative factors in healthy ecosystems and are regularly the target of eradication efforts. However, arguments against the widespread eradication of parasites have begun to emerge (Marcogliese, 2004). Firstly, in cases where parasitic infestation is common within a population, hosts have likely evolved mechanisms to tolerate it. The effects caused by, or induced from, the infestation may play a role in the normal functioning of the host immune system or physiological processes, the lack of which may lead to deleterious effects in the host (Rook, 2007; Flohr *et al*., 2009; Pizzi, 2009). As the survival of a parasite is often dependent on the (temporary) survival of the host, it is not always advantageous for the parasite to significantly affect host survival (Poulin, 2007). Secondly, as parasites have effects on host tissue, host behaviour or both, increases or decreases in parasite abundance can have significant downstream effects on both biotic and abiotic aspects of the ecosystem (Mouritsen and Poulin, 2003, 2005). As an example, parasite-induced trophic transmission (PITT), also known as trophic facilitation, is a commonly, but not ubiquitously, observed phenomenon in heteroxenous parasites (Poulin, 1994; Lafferty, 1999). By directly or indirectly enacting a change to the host, the parasite can increase the likelihood of its transmission to the following host by inducing a higher risk of predation to the host (Poulin, 1994; Lafferty, 1999). While many doubts around this process still exist, the large number of examples in its support cannot be ignored (Heil, 2016).

Box 1: A collection of applicable terms with which to describe a parasite

*Endoparasite*: Lives internally in the host, in contact with host homoeostasis.

*Ectoparasite*: Lives externally on the host, in contact with the outside environment.

*Monoxenous (direct) parasite*: One host species required for full development.

*Heteroxenous (indirect) parasite*: Multiple host species required for full development.

*Facultative parasite*: Does not require a host to complete the life cycle, but can be parasitic.

*Obligate parasite*: Requires host to complete life cycle.

*Partial parasite*: Life cycle is a combination of parasitic and free-living stages.

*Total parasite*: No free-living stages in life cycle.

Freshwater mussels have been described as keystone species for their habitats because of their effect on the nutrient dynamics of ecosystems. The filtering of suspended bacteria, phytoplankton and particulate matter, which is redeposited as larger feces or pseudofeces, provides nutrients for benthic flora and fauna, reduces water turbidity and can capture toxic elements (Vaughn and Hakenkamp, 2001; Spooner *et al*., 2013). Mussel shells can also provide physical structure as a sort of freshwater reef. Moreover, as the mussels burrow and reposition themselves, the substrate is displaced; this bioturbation increases water flow through the sediment and can create a more suitable habitat for benthic flora and fauna (Vaughn and Hakenkamp, 2001; Gutiérrez *et al*., 2003; Strayer, 2008; Spooner *et al*., 2013). The increase in macroinvertebrate densities, caused by the presence of mussel, may provide increased food sources for the host fishes, which combined with the beneficial effects of filtering on water conditions can lead to higher fish densities (Zuiganov *et al*., 1994; DuBose *et al*., 2020).

**Fig. 1.**
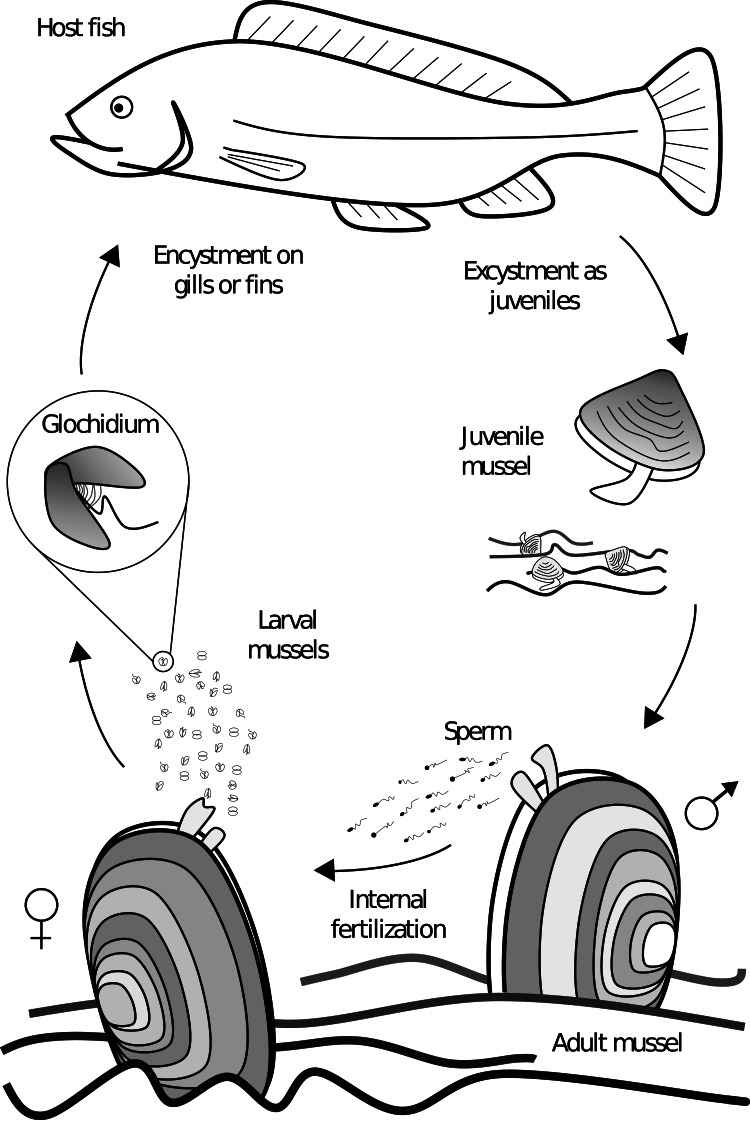
Generalized unioniform life cycle. Adult males release sperm in the water column for internal fertilization of the eggs within the female. Larvae are then released and affix themselves to the host, where they metamorphose into juveniles and detach from the host to develop into adults on the bottom substrate.

Freshwater mussels of the order Unionida are found on every continent (except Antarctica), and are characterized by the presence of a parasitic life stage (Bogan, 2008). Differing from many of their marine relatives, unionid mussels do not synchronously release eggs and sperm into the water column for their external fertilization. Unionid males release sperm in the water, which is filtered by females and used for internal fertilization (Fig. 1). In most cases, fertilized eggs are brooded in modified gill pouches for later release as parasitic larvae called *glochidia*; in some families the larvae are referred to as *lasdia* (Haag, 2012). The glochidia affix themselves to the host fish (and occasionally to amphibians) where they become encysted for a period before detaching as juvenile mussels and falling to the substrate where they bury themselves and over time develop into adults, making the mussel a partial monoxenous ectoparasite (Box 1) (Watters and O'Dee, 1996; Strayer, 2008). The location of glochidia attachment is species dependent, with some specifically encysting on gills, fins or skin, and many a combination (Arey, 1932*a*; Karna and Millemann, 1978; Silva-Souza and Eiras, 2002; Zou *et al*., 2017). Species found on gills typically encyst on the filaments and secondary lamellae, but they can also be found on gill arches, rakers and pseudobranchs (Karna and Millemann, 1978; Waller and Mitchell, 1989). The parasitic life stage allows for mussel growth and dispersal, in particular upstream dispersal with host movement in streams and rivers (Wächtler *et al*., 2001; Korniushin and Glaubrecht, 2003; Graf, 2013). Given the specific fitness advantage of this parasitic life-history adaptation, the Unionida order is almost exclusive to moving fresh water, with only a few species known to tolerate brackish water (Wächtler *et al*., 2001; Korniushin and Glaubrecht, 2003; Haag, 2012; Graf, 2013).

Unionid mussels are a highly endangered group of organisms due to widespread habitat loss, environmental degradation and overfishing (Buddensiek *et al*., 1993; Bauer and Wächtler, 2001; Cosgrove and Hastie, 2001; Skinner *et al*., 2003; Bogan, 2008; Österling *et al*., 2008, 2010; Lundberg and Österling, 2016; Lopes-Lima *et al*., 2017). These threats have led to massive worldwide declines in unionid populations, with 37 species presumed extinct in North America alone and most others classified as at least threatened (Bogan, 2008). The International Union for Conservation of Nature (IUCN) classifies freshwater molluscs as the most highly endangered group of organisms in Europe (IUCN, 2015). As ectoparasites, glochidia are particularly sensitive to negative environmental changes as both the host and the parasite are exposed to environmental degradation, thus compounding any potential negative effect caused by environmental change (Hairston and Bohonak, 1998; Poulin, 2007). Reintroduction strategies and conservation research are therefore fundamental for the survival of the species in this order and to the function of their associated ecosystems (Skinner *et al*., 2003; Bogan, 2008; DuBose *et al*., 2020).

Unionid mussels are an understudied order. While other bivalve orders share similarities in basic biology and ecosystem services, unionids are unique with their parasitic life history. A recently published review by Modesto *et al*. (2018) showed the importance of fish for freshwater mussel conservation. This review, on the other hand, aims to specifically summarize the current literature set on the impacts parasitic mussels have on behaviour and physiology of host fishes. The present synthesis will facilitate effective reintroduction and conservation actions, as well as spur future research. In doing so, gaps in the literature will be identified and suggestions for future investigations to fill them will be proposed. This work is of particular importance as the endangered nature of this order has only recently begun being addressed and the lack of deep understanding prevents preservation efforts from reaching high efficacy.

## Literature overview

### Literature search

Three literature searches were performed on 20 September 2021 in both Web of Science (Karlstad University library subscription) and Google Scholar with the following search strings: ‘ALL = ((glochid* OR mussel larv* OR parasitic mussel OR margaritifera OR unio) AND (effect OR causes OR impairs OR improves OR increases OR decreases) AND (host OR fish OR salmon OR trout OR bass OR salmonid OR minnow OR darter))’, which returned 786 and 72 hits, respectively. A third search on Google Scholar on the same date with the search strings: ‘glochidia’ ‘effect on host’ returned 39 hits.

Of the 897 total hits recovered, an initial selection was made based on their title and abstract to exclude studies with no relevance to unionid mussels. Papers on differential host suitability to glochidia were excluded, because this did not relate to our focus of behavioural and physiological impacts of glochidia on the host. A later reading of the retained papers resulted in 35 studies being classified as relevant for the review as they investigated the direct impact of glochidia infestation (glochidiosis: the disease of having glochidia) on host behaviour and/or physiology, including 1 publicly available master thesis. When PhD dissertations were found to be of relevance, the specific manuscript or chapter of interest was identified and extracted. Further, the reference list and cited-by list of all 35 papers were investigated for additional relevant studies not discovered by the search strings; this revealed 28 additional studies, including another publicly available master thesis, through a similar screening process as before. Five public access bachelor and master thesis reports from within Karlstad University not revealed by the literature searches were also included as they investigated the impact of glochidia on host behaviour and/or physiology. A collective 69 titles were retained after excluding studies with no relevance to our review (Supplementary Table S1).

### Publication characteristics

**Fig. 2.**
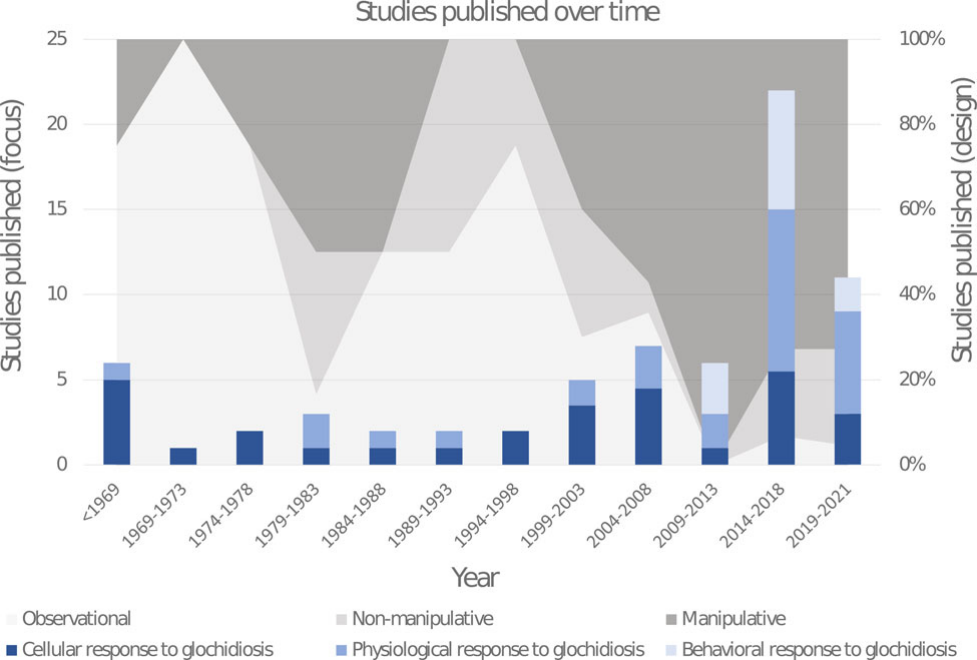
Distribution histogram of the publication dates of papers investigating the impact glochidiosis has on behaviour and physiology of host fishes. Specific paper focus is represented in blue within the histogram bars (dark blue: cellular, mid-blue: physiological, light blue: behavioural). The proportional usage of different study designs through time are represented with a shaded background (light grey: observational, mid-grey: non-manipulative, dark grey: manipulative). Papers with multiple foci, or using multiple designs were recorded with 0.5 or 0.33 in their respective counts to result in a total sum of 1 per paper. Six papers published between 1919 and 1942 were grouped into 1 year range labelled <1969.

Approximately half of the recovered papers were published within the last 10 years (Fig. 2). The studies were classified according to their different response foci (cellular, physiological or behavioural). Studies on the cellular responses to glochidiosis include histological and immunological investigations and primarily encompass cyst formation and host resistance to infestation, and have historically been the most popular area of research. Studies on the physiological responses to glochidiosis include investigations on all other physical changes measured on host biology, which were divided into the following 8 categories: whole body (e.g. growth rate and survival), metabolic rate, organ effects, toxicology, gene expression, colouration, reproduction and molecular changes. Studies on the behavioural responses to glochidiosis have only begun to appear in the last decade and included all investigations involving host activity levels, feeding rates, habitat preferences, migration and social interactions; roughly 30% of these behavioural studies are comprised of grey literature. Where possible, numerical results were extracted for more in-depth comparisons.

Studies were also classified according to their study designs (manipulative, non-manipulative or observational). Manipulative studies compared effects on fish induced by artificial infestation of glochidia with controls without infestation. This experimental design has become the norm for unionid research around the turn of the century. Non-manipulative studies related to potential effects induced by glochidia infestation, did not specifically manipulate glochidial load. These were isolated from manipulative studies, as they investigate correlative patterns of infestation rate and not causal relationship; i.e. did the glochidia cause the effects or did the measured effect or traits associated with the measured effect influence glochidia susceptibility. Observational results did not numerically compare an effect with controls, but rather verbally described noted changes, generally over time. Some papers included investigations with more than 1 study design or focus; these were placed in all relevant categorizations.

**Fig. 3.**
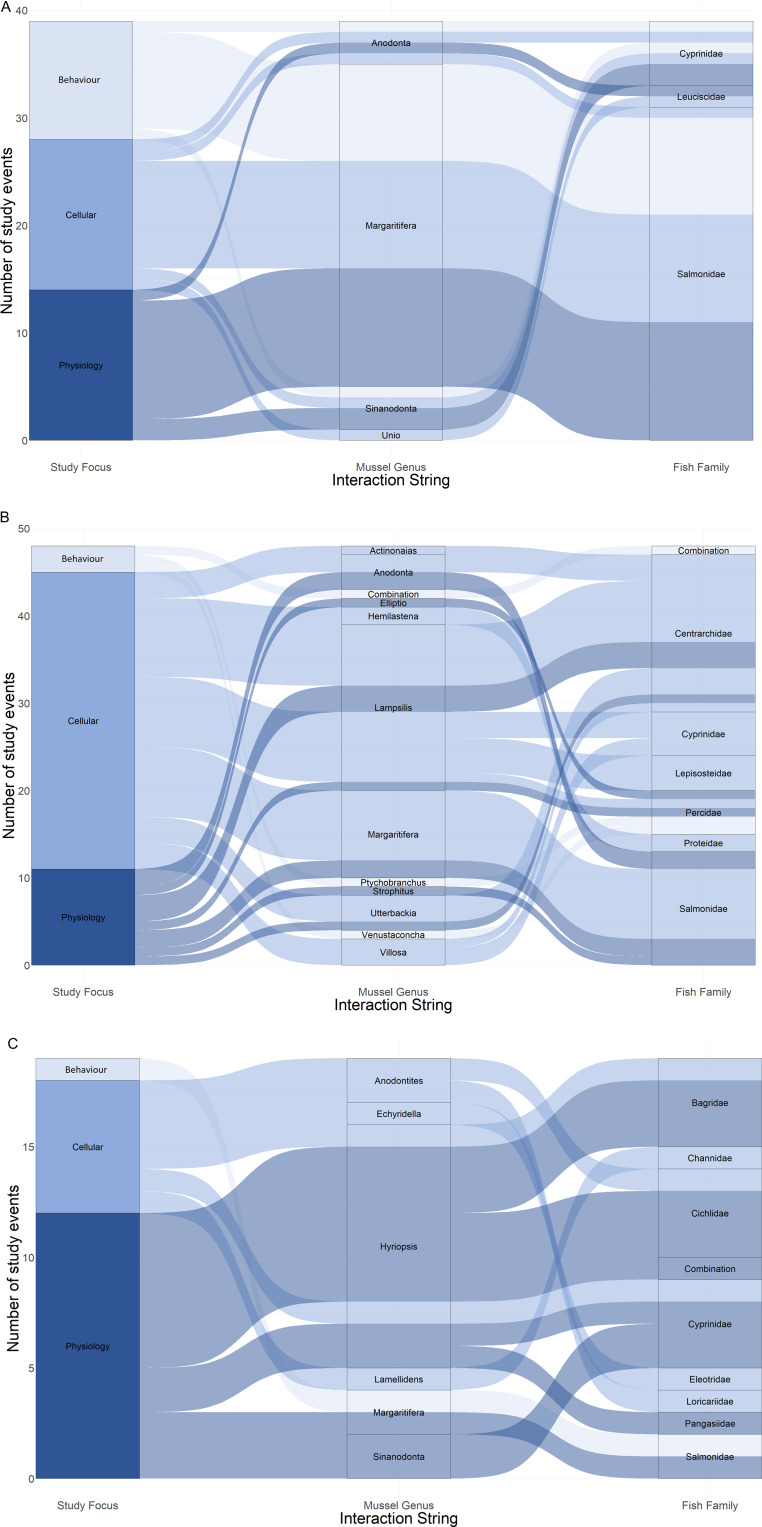
Alluvial plots showing proportional representation of study focus per mussel genus and fish family, listed alphabetically, separated by continent of origin: (A) Europe, (B) North America and (C) other.

Species representation between the papers was skewed. Of the 29 mussel species included in this review, over half were only investigated once, whereas *Margaritifera margaritifera* was included in 28 papers (41%). Similarly, of the 46 fish species studied, almost half were only investigated once, whereas *Salmo trutta* was included in 17 papers (37%, Fig. 3). Consequently, the most represented species interaction was that between *M. margaritifera* and *S. trutta*, investigated 22 times (20%, different foci counted separately, 110 individual results), whereas 19 combinations of mussel and fish species were only investigated once (17%).

## Cellular responses to glochidiosis

Within the 33 studies on the cellular response to infestation, 3 major categories were identified: cyst formation and associated histopathology, resistance to infestation and acquired immunity to infestation. Given the largely descriptive nature of the majority of the studies with this focus, numerical result extraction and analysis was not performed. This focus area has been investigated the longest with the first descriptions dating back to 1919 (Reuling, 1919).

### Cyst formation

After contact with an appropriate host tissue, the glochidia ‘bites’ down causing trauma to the upper epithelial membrane and engulfing deeper host tissue (Arey, 1932*a*). Different glochidia morphologies (hooked *vs* hookless) cause different degrees and variants of trauma (piercing *vs* shearing), though firmer underlying structural tissue generally appears unaffected (Arey, 1932*a*; Karna and Millemann, 1978; Waller and Mitchell, 1989). Attachment does not typically cause damage to the underlying blood vessels or result in haemorrhaging or seepage, though the clamping force can restrict blood flow (Arey, 1932*a*; Karna and Millemann, 1978; Meyers *et al*., 1980; Howerth and Keller, 2006). While uncommon, initial attachment has been associated with widespread haemorrhaging and necrosis of host tissue that can lead to near immediate host mortality (Howerth and Keller, 2006).

**Fig. 4.**
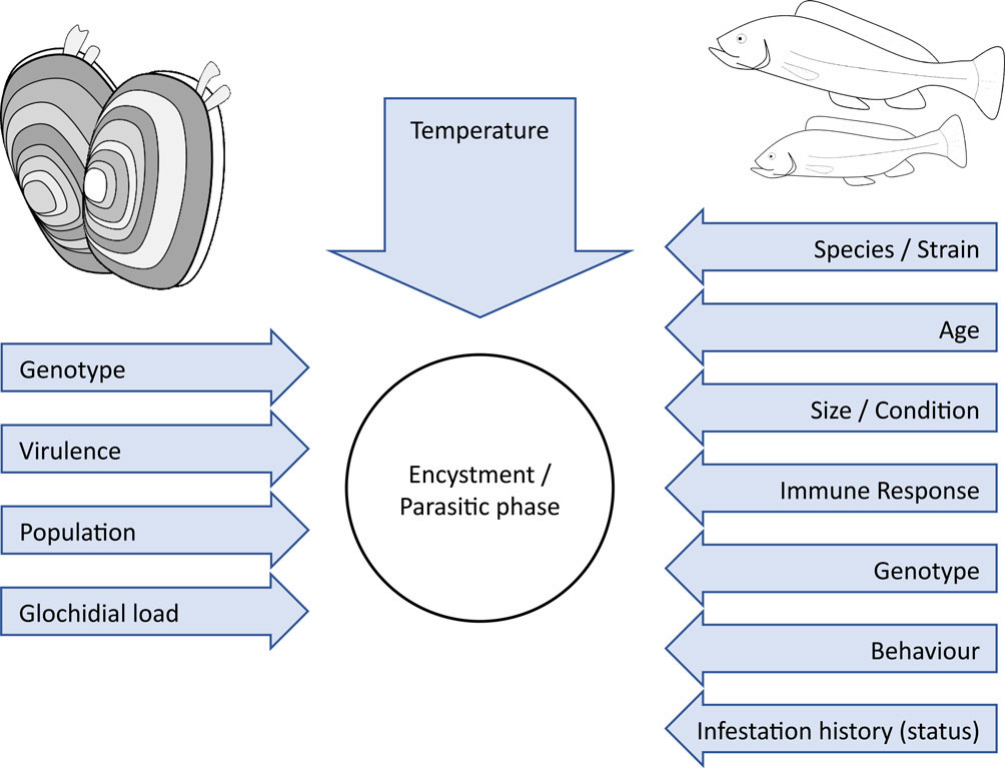
Host, parasite and environmental factors that can have an influence on the Unionida–fish host–parasite interaction. Redrawn from Marwaha (2020).

Glochidia infestation on gills can induce asphyxia by reducing blood flow, surface area for gas exchange and optimal water flow over lamellar tissue (Karna and Millemann, 1978; Howerth and Keller, 2006; Castrillo *et al*., 2019). Many small lesions to the gill tissue is a particularly harmful stressor as the high vascularization and constant contact with the outside environment lead to the rapid efflux and/or influx of pathogens, ions and other molecules, thereby increasing osmotic and immunological stress (Karna and Millemann, 1978; Quilhac and Sire, 1999; Silva-Souza and Eiras, 2002; Howerth and Keller, 2006; Castrillo *et al*., 2019). Rapid wound healing through cellular migration is a common non-specific response to gill damage, seen both in response to other gill parasites and in tissue lesions (Arey, 1921; Paperna, 1964; Quilhac and Sire, 1999; Adams and Nowak, 2001; Ferguson and Speare, 2006; Matthews *et al*., 2013). Host cellular migration over the glochidial body causes cyst formation, as can be seen by the presence of goblet, pigment and epithelium cells, along with host connective tissue in the cyst wall (Arey, 1932*a*; Nezlin *et al*., 1994; Rogers-Lowery and Dimock, 2006; Castrillo *et al*., 2019). Cyst formation has been demonstrated to be a non-specific response as lesions from metal chips lodged in gill tissue cause a similar encystment process (Arey, 1921). Cysts can completely cover the larvae within 2 h (Arey, 1923; Rogers-Lowery and Dimock, 2006). The exact rate of cyst formation is highly dependent on a variety of factors such as temperature (Taeubert *et al*., 2014), host suitability (Waller and Mitchell, 1989), prior to host exposure to glochidia infestation (Rogers-Lowery and Dimock, 2006) and can even differ between individual glochidia as the process is not synchronous for all attached glochidia, even those encysted in the same vicinity (Nezlin *et al*., 1994; Rogers-Lowery and Dimock, 2006) (Fig. 4).

When a glochidia attaches to a gill filament, cyst growth can cause extensive fusion of lamellae (Fustish and Millemann, 1978; Karna and Millemann, 1978; Waller and Mitchell, 1989; Treasure and Turnbull, 2000; Castrillo *et al*., 2019). In cases of extreme parasitic load, the extensive fusion of lamellae can obliterate all finer structures, giving the filament a smooth outline (Howerth and Keller, 2006). When a cyst forms on the distal end of the filament, the tip often curls giving it a club-like appearance (Karna and Millemann, 1978). Encysted lamellae can be different in size to un-encysted ones, an effect that can persist even after excystment (Kaiser, 2005; Thomas *et al*., 2014). Reduced osmotic ability and gas exchange rates can, but do not always, persist after the death or excystment of glochidia; there appears to be a connection to initial encystment load (Treasure and Turnbull, 2000; Kaiser, 2005; Castrillo *et al*., 2019; Horne, 2021).

No specific glochidia structures have been observed to extract nutrients from the host, but stable isotope analysis does show such transfer (Arey, 1923*a*, 1923*b*; Fritts *et al*., 2013; Denic *et al*., 2015). It is hypothesized that this transfer results from the glochidia digesting the host tissue captured within the initial bite (Arey, 1923*a*, 1923*b*; Fritts *et al*., 2013; Denic *et al*., 2015). Digestive enzymes used in the process of breaking down the captured tissue may seep out of the glochidia and digest some surrounding host tissue. Passive absorption of compounds from the intercellular space and blood plasma has also been proposed (Arey, 1923*a*, 1923*b*; Fritts *et al*., 2013; Denic *et al*., 2015). Blystad (1923) suggested that, as blood continues to flow through the host tissue, a ‘placenta-like’ relationship develops between the host and the glochidia. Arey (1923*a*) noted that only ‘*Proptera* glochidia type’, which undergo a large size increase in the post-metamorphic retention period, display capillary growths in the ‘very large and thick’ cyst wall. In conclusion, the process of cyst formation is well described, although the factors affecting it and the long-term cellular impacts remain poorly understood.

### Histopathology of glochidiosis

Upon encystment, host tissue typically responds with widespread hyperplasia, hypertrophy, spongiosis and sloughing; or, increase in cell count, cell size, intracellular spaces and epithelial shedding (Arey, 1932*a*; Fustish and Millemann, 1978; Meyers *et al*., 1980; Waller and Mitchell, 1989; Treasure and Turnbull, 2000; Castrillo *et al*., 2019). Sloughing is a common mechanism for the removal of epidermal pathogens; this typically refers to the shedding of the upper mucous membrane, but can also include the upper epithelial tissue (Ángeles Esteban, 2012). Sloughing of encysted glochidia occurs through an initial thinning of the cyst wall in a process loosely described as the reverse of cyst formation; the mechanical force of the water flowing over the gills then forces the larva off the tissue (Arey, 1932*c*). Occasionally, a cyst can become stalked, with only a thin layer of cells engulfing the parasite held to the host *via* a thin filament of host cellular tissue making it more easily removed (Arey, 1932*c*; Meyers *et al*., 1980; Watters and O'Dee, 1996). The impacts of glochidiosis on the mucous cell counts on the gills of infested hosts vary; Treasure and Turnbull (2000) showed an increase whereas Thomas *et al*. (2014) showed a decrease, likely a result of glochidia being observed at different time points.

Studies investigating the long-term histopathology of glochidiosis are sparse and mostly restricted to *M. margaritifera*, as relatively few mussel species have encystment periods longer than several weeks. After approximately 14 days, hypertrophy and hyperplasia in hosts infested with *M. margaritifera* become reduced and localized around the glochidia (Treasure and Turnbull, 2000; Wang *et al*., 2016; Castrillo *et al*., 2019).

When discussing cyst formation, many authors note increases in tissue hyperplasia, this is, however, a misnomer, as in the modern lexicon, the term ‘hyperplasia’ refers to an increase in cell count *via* cell proliferation, not migration (Petersen, 2007; NIH, 2021). Generally, no significant increase in mitotic events is described during the early stages of glochidiosis, though some reports do exist (Arey, 1932*b*; Waller and Mitchell, 1989; Nezlin *et al*., 1994; Rogers-Lowery and Dimock, 2006; Castrillo *et al*., 2019). While a later period of cell proliferation likely occurs to replace lost cells and restore prior tissue configuration, mitotic events are not the primary drivers of cyst formation (Arey, 1932*b*; Nezlin *et al*., 1994; Rogers-Lowery and Dimock, 2006; Castrillo *et al*., 2019). On the other hand, no specific term exists to differentiate between an increase in cell count *via* migration or proliferation.

### Resistance to infestation

In host fish, the immune response against glochidial infestations is generally divided into innate (also referred to as natural, racial or non-specific) (Reuling, 1919; Arey, 1932*c*; Donrovich *et al*., 2017) and adaptive (also referred to as acquired or delayed) immunity (Watters and O'Dee, 1996; Dodd *et al*., 2006). Innate immunity acts against glochidia in a generalized and non-specific manner, rapidly killing or removing glochidia on incompatible hosts (Donrovich *et al*., 2017). Adapted immunity, on the other hand, begins to develop after a first infestation event and becomes more effective at protecting the host after repeated infestation events on compatible hosts (Dodd *et al*., 2006). In innate immunity, encysted glochidia can be completely removed in as little as 2 days (Arey, 1932*c*). Initial cyst growth on naturally immune (non-compatible) hosts appears similar to the cyst growth on compatible hosts, although the final cyst forms more slowly and becomes fibrous, thicker and more irregular (Reuling, 1919; Arey, 1932*c*; Watters and O'Dee, 1996; Rogers-Lowery and Dimock, 2006). Generally, there is a negative relationship between the period of cyst formation and glochidia success rate indicating the presence of a persistent anti-glochidia mechanism (Arey, 1932*a*; Nezlin *et al*., 1994).

In both cases, inflammatory granulocyte infiltrates can be observed in the areas afflicted by the infestation within the first hours of attachment (Waller and Mitchell, 1989; Watters and O'Dee, 1996; O'Connell and Neves, 1999; Treasure and Turnbull, 2000; Dodd *et al*., 2006; Rogers-Lowery *et al*., 2007; Castrillo *et al*., 2019). In suitable hosts, these infiltrates are associated with the initial termination of many glochidia within the first weeks (Castrillo *et al*., 2019). Through intraperitoneal injections of cortisol, an immunosuppressant, Dubansky *et al*. (2011) were able to increase successful glochidial metamorphosis, as a product of higher initial glochidial encystment success. In naturally immune and less suitable hosts, a different pathogenesis is likely at play as the complete (or near-complete) termination of all glochidia occurs over the course of days rather than weeks (Waller and Mitchell, 1989; Treasure and Turnbull, 2000; Castrillo *et al*., 2019). Kirk and Layzer (1997) induced glochidia to metamorphose on a naturally immune host, again with cortisol as an immunosuppressant.

As the case with histopathology, studies investigating the long-term immune effects of glochidiosis are sparse and mostly restricted to *M. margaritifera*. After approximately 14 days the inflammatory infiltrates shift in composition to a lymphocytic stage, in line with the responses observed in other tissue parasites (Treasure and Turnbull, 2000; Wang *et al*., 2016; Castrillo *et al*., 2019).

### Adaptive immunity

Adaptive immunity to glochidiosis is highly species specific, with high intraspecific variability (Reuling, 1919; Arey, 1923; Dodd *et al*., 2006). Two generalized forms of adaptive immunity appear to manifest, classified based on the effect observed on the parasitic larvae, and are referred to here with the novel terms: hard [-acquired] immunity and soft [-acquired] immunity which are likely associated with the *cell-mediated* or *antibody* immune responses (Raff *et al*., 2002). Hard immunity appears to improve the initial host immune response, resulting in a lower level of initial infestation, and in some cases fending the infestation off completely through a cell-mediated immune response (Reuling, 1919; Arey, 1923; Dodd *et al*., 2006; Chowdhury *et al*., 2017; Donrovich *et al*., 2017). Soft immunity differs from hard immunity as glochidial load is similar to that of un-primed hosts, but appears to result in maintained glochidia antagonism, ultimately leading to an extended sloughing period of dead or un-metamorphosed glochidia, as well as an early release of lower quality metamorphosed glochidia through an antibody immune response (Reuling, 1919; Arey, 1923; Bauer and Vogel, 1987; Watters and O'Dee, 1996; Rogers and Dimock, 2003; Dodd *et al*., 2006; Treasurer *et al*., 2006; Chowdhury *et al*., 2017). Donrovich *et al*. (2017) suggest that reductions in fish health status, among other measures, after multiple infestations may also play a role in reduced glochidial susceptibility.

Soft immunity can be seen to form in hosts after as little as 1 infestation (Bauer and Vogel, 1987; Dodd *et al*., 2006; Treasurer *et al*., 2006; Zou *et al*., 2017). On the other hand, hard immunity seems to take multiple infestations to develop and appears to be dependent on the length of the infestation period. For mussels with an infestation period measured in weeks, it may take as little as 2 previous infestations (priming events) to develop hard-acquired immunity (Dodd *et al*., 2006), whereas it may take up to 6 infestation events for mussels with shorter infestation periods (Seshaiya, 1969). Reports stating the absence of acquired immunity do not extensively re-infest hosts or measure the quality of glochidia from later infestations and should therefore be evaluated in that light (Young *et al*., 1987; Chowdhury *et al*., 2017; Hanrahan, 2019). Infestation intensity has no clear effect on the acquisition of immunity, with Arey (1923) describing no effect, but Chowdhury *et al*. (2017) the contrary. High variation between individual hosts has also been a noted factor in the acquisition of immunity (Arey, 1923). Moreover, infestation from 1 mussel species can influence the metamorphosis success of another (Reuling, 1919; Arey, 1923; Rogers and Dimock, 2003; Dodd *et al*., 2005; Chowdhury *et al*., 2017; Donrovich *et al*., 2017). The duration for which immunity is retained is unknown, but reports suggest that hard immunity is lost relatively quickly whereas soft immunity can last for at least a year (Arey, 1923; Dodd *et al*., 2006).

Studies of cross-parasite immunity have shown mixed results. One study reports that hosts recently infested with glochidia have a higher predisposition to the eye fluke *Diplostomum pseudospathaceum*, also a gill-targeting parasite (Gopko *et al*., 2018). Likewise, hosts recently infested with the eye fluke were also more predisposed to glochidia infestation (Gopko *et al*., 2018). On the other hand, another study suggests that *S. trutta* infested with glochidia had a marginal increase in life expectancy when infected with a highly virulent and terminally fatal strain of the bacterium *Flavobacterium columnare*; an effect noticeable both at 2 and 14 months post-infestation; the mechanisms remain unknown (Chowdhury *et al*., 2021). The long-term effects of infestation, particularly those relating to immunity, are poorly understood and deserve attention in future work.

## Physiological responses to glochidiosis

Within the 30 studies on physiology, 410 individual results were extracted and assigned to 8 broad categories, which were further divided into 25 sub-categories (Table 1). To be included in our review, a statistical output should have been reported within the text or a figure. Statement such as ‘no difference in mortality between infested and non-infested fish’ was considered valid results showing no effect. In rare instances of multi-way analyses of variance, where no *P*-value for a specific main or interaction effect was given, the ±1 standard error bars of the given plots were compared, if they overlapped it was considered a no-effect result (Supplementary Table S2). The most well supported of the result categories are the ones related to whole-body effects, molecular changes and metabolic rate effects, supported by 19, 10 and 9 studies, respectively.

**Table 1.** Reported results of specific physiological effects experienced by host fishes during and shortly after glochidiosis

Category	Papers represented	Mussel species represented	Fish species represented	Sub-category	Lower	No change	Higher	Papers represented	Mussel species represented	Fish species represented
Whole body	19	8	8	Growth	13	15	0	13	5	6
				Length	3	12	0	6	2	3
				Condition factor (*k*)	4	13	3	8	3	4
				Survival	6	9	4	9	4	4
Metabolic rate	9	8	8	Oxygen consumption	12	38	4	8	8	7
				RI	0	18	0	1	1	2
				Tolerance to hypoxia	8	18	0	2	2	2
				EMG readings	1	2	3	1	1	1
				Ammonia excretion	0	0	2	1	1	1
Organ histology	3	3	3	Spleen size	0	4	1	2	2	2
				Liver size	0	1	0	1	1	1
Toxicity	2	2	2	LD-50	6	0	0	1	1	1
				Bioaccumulation	0	2	0	1	1	1
Gene expression	2	3	2	Immune function	2	0	0	1	1	1
				Malignant tumours	0	0	1	1	1	1
				Stress response	3	12	2	2	3	2
Colouration	2	2	2	Colouration	0	1	1	2	2	2
Reproduction	1	1	1	Reproductive potential	3	1	0	1	1	1
Molecular changes	10	5	10	Haemoglobin	2	7	2	5	3	3
				Hormones	0	4	4	4	3	4
				Sugars	0	23	0	4	3	8
				Free fatty acids	5	12	1	3	2	7
				Free enzymes	0	2	6	2	1	2
				Proteins	4	27	2	3	2	6
				Dissolved ions	4	67	10	5	4	8

Further information as to the number of studies and species across which the results are described across are also reported.

Whole-body effect is an important category, as it represents a general set of results providing researchers with an easy and effective way to overview the general effects of infestation. Species representation in this category was highly skewed; almost 80% of all studies focused on the relationship between *Margaritifera* spp. and Salmonidae, with only 2 studies not investigating the effect of *M. margaritifera* on either *S. trutta* or *Salmo salar* (Table 1). Fifty-two per cent of the results in this category were manipulative, with 42% non-manipulative and 6% observational.

Metabolic rate is a highly relevant category of results to investigate in the Unionida–host system because larvae commonly encyst on gills, thereby directly influencing oxygen uptake capacity. The species representation in this category had a much more even species distribution than studies on whole-body effects, but was investigated in half as many studies (Table 1). The *M. margaritifera*–*S. trutta* interaction was studied only twice, the divergent action of 1 mussel species was studied once; the differential impact of separate mussel species on a fish was studied twice. Eighty-four per cent of the results in this category were manipulative, with the remaining 16% non-manipulative; no results in this category were observational.

Investigations on molecular changes provide valuable insight into the mechanisms employed by hosts to tolerate infestation stress. This category was evaluated in 10 studies, roughly the same as metabolic effects, with, however, twice the host species count (19) as mussel species count (5), providing valuable information of the potential divergent action of different parasitic mussels. The results in this category were evenly split between manipulative and non-manipulative, with no observational results. All reported measures below were from blood plasma unless otherwise specified.

Collectively, the combination of all other categories did not demonstrate a prominent species skewness as only 2 of the 7 investigated the *M. margaritifera*–*S. trutta* interaction. This combination of categories investigates the impact of glochidiosis on organ histology, toxicology, gene transcription, colouration and mating. Of the 39 results only 1 was non-manipulative, with all others possessing a manipulative design.

### Whole-body effects

Overall, infestation from *M. margaritifera* has shown broad differences in effect on *Salmo* spp. at different infestation levels. Glochidial load appears to be particularly important in determining effect direction, exemplified through the comparison of 2 recently published studies on hatchery-reared lab-infested 1-year-old (1+) brown trout (Chowdhury *et al*., 2019; Marwaha *et al*., 2019). The first study reports a negative effect on growth at 84, 203, 266 and 315 days post-infestation (dpi) when infested with an initial load of ~5000 glochidia per fish (gl/f) compared to controls, with no difference in mortality (Chowdhury *et al*., 2019). The second indicated no manipulative or non-manipulative difference in mass, length or mortality of 1+ brown trout at 300 dpi when infested with an initial load of ~213 gl/f (Marwaha *et al*., 2019). Moreover, the second study outlined a positive impact on host condition factor with both a manipulative and non-manipulative interpretation of the results (Marwaha *et al*., 2019).

There is an incongruity between authors regarding the time frame across which negative effects in salmonids infested with *M. margaritifera* are observed (Bruno *et al*., 1988; Cunjak and McGladdery, 1991; Treasure and Turnbull, 2000; Zuiganov, 2005; Treasurer *et al*., 2006; Taeubert and Geist, 2013; Filipsson *et al*., 2016; Freitt, 2016; Andersson, 2018; Chowdhury *et al*., 2019; Marwaha *et al*., 2019). While negative effects on trout growth and survival within the first month (<30 dpi) have been noted (Taeubert and Geist, 2013; Andersson, 2018), this is not consistently reported in all studies on the topic; growth rate is often reduced but not condition factor (Treasurer *et al*., 2006; Freitt, 2016; Andersson, 2018). Within the early infestation period (⩽100 dpi), negative effects on fish weight, growth rate and condition factor have been demonstrated using both manipulative and non-manipulative study designs (Treasurer *et al*., 2006; Freitt, 2016; Chowdhury *et al*., 2019), although these effects have not always been observed (Filipsson *et al*., 2016; Freitt, 2016; Andersson, 2018; Marwaha *et al*., 2019). Results reporting the effects on the mid (>100 to <200 dpi) and late (200+ dpi) infestation periods are mixed regarding growth, mass, length and condition factor, similarly to the results reported in the early infestation period (Bruno *et al*., 1988; Cunjak and McGladdery, 1991; Treasure and Turnbull, 2000; Treasurer *et al*., 2006; Filipsson *et al*., 2017; Chowdhury *et al*., 2019; Marwaha *et al*., 2019). The reported inconsistent effects are complemented by reports suggesting infestation could improve both the condition factor and resistance to trauma of host fishes (Zuiganov, 2005; Marwaha *et al*., 2019).

Mussels with shorter encystment periods are more likely to cause negative effects on host growth (10/17 results are negative from 7 studies) than *M. margaritifera* (15/65 results are negative from 12 studies), particularly at high infestation levels (Supplementary Table S2; Murphy, 1942; Moles, 1983; Crane *et al*., 2011; Du *et al*., 2015; Douda *et al*., 2017). At low levels of infestation, less, if any, negative effects on these same measures have been observed (Crane *et al*., 2011; Douda *et al*., 2017; Defo *et al*., 2019; Methling *et al*., 2019). Ooue *et al*. (2017) reported no change in the host growth rate during the infestation period but did report a negative growth rate in the post-infestation recovery.

### Metabolic rate

*Margaritifera margaritifera* has a negative impact on the metabolic rate of *S. trutta* (Thomas *et al*., 2014; Filipsson *et al*., 2017). At 160 dpi, lab-infested hatchery-reared young-of-the-year (0+) *S. trutta* exhibited an increased ventilation rate compared to a control; furthermore, infestation intensity was positively correlated with ventilation rate within the treatment group (Thomas *et al*., 2014). At ~250 dpi, wild-caught, wild-infested 1+ trout displayed higher standard metabolic rate (SMR) and maximum metabolic rate than their wild-caught uninfested controls (Filipsson *et al*., 2017). On the other hand, SMR was significantly negatively correlated with infestation rate, making the more heavily infested individuals appear more similar to the average control SMR (Filipsson *et al*., 2017). This effect was hypothesized to be a result of additional physiological effects at high loads countering the effects induced by glochidiosis (Seppänen *et al*., 2009; Filipsson *et al*., 2017).

Studies on host metabolic activity show mixed, but mostly negative short-term effects on host oxygen consumption, muscle activity and ammonia excretion (Du *et al*., 2015; Slavík *et al*., 2017; Methling *et al*., 2018, 2019). No difference in baseline oxygen consumption (MO_2_) was observed in *Rhodeus ocellatus* compared to controls at <1 dpi, despite being significantly reduced after the administration of a stressor. Likewise, *Rhodeus amarus* exhibited a significantly lower ΔSMR before and after glochidia infestation treatment than controls (subjected to sham infestation) at 1 dpi; this difference disappeared at 2 and 3 dpi but returned as a significantly higher ΔSMR at 4 dpi (Methling *et al*., 2018). This delayed effect was speculated to be a secondary effect from the cortisol release after initial encystment disrupting the hydromineral balance and disturbing the intermediary metabolism (Douda *et al*., 2017; Methling *et al*., 2018). On the other hand, no change in MO_2_ of *Pelteobagrus fulvidraco* was observed at 12 dpi during a manipulative study, though infestation did significantly increase ammonia excretion, a difference also correlated with glochidial load (Du *et al*., 2015). Electromyogram (EMG) readings in *Cyprinus carpio* at <4 dpi were higher in relation to controls, whereas the difference in EMG readings at 8 dpi were only significantly different during the day, but not in the night (Slavík *et al*., 2017).

Investigations on ventilation rate and oxygen consumption suggest that hosts may not tolerate infestation from 2 mussel species to the same degree (Kaiser, 2005; Crane *et al*., 2011; Horne, 2021). The ventilation rate of *Etheostoma caeruleum* infested with 2 mussel species was unaffected when calculated as a 14 dpi average. However, host ventilation was significantly higher than controls early in the *Venustaconcha pleasii* infestation but later during the *Ptychobranchus occidentalis* infestation period (Crane *et al*., 2011). Similarly, the metabolic activity of *Micropterus salmoides* responded differently to infestation from *Lampsilis straminea* and *Lampsilis reeveiana*, though the degree to which this effect is dominated by infestation intensity and not species is unclear (Kaiser, 2005; Horne, 2021). When infested with ~632 gl/f of *L. reeveiana*, hosts displayed higher ventilation, lower MO_2_ and critical dissolved oxygen (DO_crit_) at every point of a 13-week observation period when compared to controls; these differences were all significantly correlated with non-manipulated glochidial loads (positive, negative, negative) (Kaiser, 2005). On the other hand, no differences in routine metabolic rate, regulation index (RI) or DO_crit_ were observed at any point during an 11-week observation period when infested with a quarter the glochidial load of *L. straminea* (~150 gl/f, Horne, 2021).

### Molecular changes

Studies focusing on molecular changes in *M. margaritifera* have shown little to no effect on glochidia infestation (Treasure and Turnbull, 2000; Thomas *et al*., 2014; Filipsson *et al*., 2017; Marwaha *et al*., 2019). No differences in haematocrit counts have been observed (Thomas *et al*., 2014; Marwaha *et al*., 2019), though 1 non-manipulative study reports it correlated with glochidial loads in wild-caught trout (Filipsson *et al*., 2017). Infestation appears to reduce the homoeostatic capacity of *S. salar* (Treasure and Turnbull, 2000).

Studies on glochidiosis of the invasive *Sinanodonta woodiana* in multiple hosts indicate that, while host osmotic ability and liver function may be affected by infestation, it likely does not cause increased stress to *C. carpio* or *Squalius cephalus* (Douda *et al*., 2017; Slavík *et al*., 2017). In both hosts, chloride, potassium, aminotransferase and alanine aminotransferase concentrations were positively related to the level of infestation treatment (Douda *et al*., 2017; Slavík *et al*., 2017). Conversely, calcium, sodium and cortisol were reported as unchanged in both hosts (Douda *et al*., 2017; Slavík *et al*., 2017). Concentrations of alkaline phosphatase and lactate dehydrogenase, enzymes that are commonly elevated when the liver is damaged, did not change in *C. carpio*, but were reported as elevated in *S. cephalus* (Douda *et al*., 2017; Slavík *et al*., 2017). No changes in haematocrit or haemoglobin concentrations were reported in *C. carpio* (Slavík *et al*., 2017).

Two studies on the impacts of *Hyriopsis cumingii* infestation on the nutritional value of its hosts demonstrate that glochidiosis has little to no effect on host sugar, fatty acid, protein or amino acid concentrations in plasma, liver or muscle tissue during or after infestation (Wen *et al*., 2009; Ma *et al*., 2018). There were however some effects. *Pelteobagrus fulvidraco* experienced a significant dip in plasma total amino acid scores from 5 dpi onwards, whereas *Oreochromis niloticus* had reduced triglyceride and low-density lipoprotein scores only after the glochidia had dislodged, potentially indicating chronic stress (Wen *et al*., 2009; Ma *et al*., 2018).

One study focusing on the nutritional requirements of developing glochidia indicates that *Hyriopsis myersiana* has little to no effect on host amino acid concentrations, fatty acid, total protein or mineral concentrations in blood plasma (Uthaiwan *et al*., 2003). A cross-species average between *C. carpio*, *O. nilotica*, *Pangasius pangasius* and *Clarias macrocephalus* × *Clarias gariepinus* hybrid shows an overall increase of total plasma protein in both the early and late stages of infestation (3 and 12 dpi) with respect to controls; some further differences in mineral and amino acid levels were also significantly different than controls (Uthaiwan *et al*., 2003). In isolation at 12 dpi, *C. carpio* was completely unaffected by infestation while the other 3 species displayed reduced triglyceride concentrations and some heightened mineral levels. All 4 species exhibited minor changes in some free amino acid levels (Uthaiwan *et al*., 2003).

*Utterbackia imbecillis* induces no major changes in hosts indicative of energetic or osmotic imbalances or consistent effects to blood-oxygen transport at 1 dpi with multiple levels of infestation (Dubansky *et al*., 2011). On the other hand, cortisol was significantly increased for all glochidial loads above the lowest indicating that heavier infestations are more stressful to hosts than lighter infestations (Dubansky *et al*., 2011).

### Other categories

Infestation generally causes no change in either liver or spleen morphology (Thomas *et al*., 2014; Douda *et al*., 2017; Defo *et al*., 2019). However, 1 study on the *M. margaritifera*–*S. trutta* interaction noted spleen size to be increased in relation to controls at 30 dpi, but not at 15 or 160 dpi (Thomas *et al*., 2014).

Infestation does not appear to cause significant changes in stress-related gene transcription rates when compared to other stressors, although some genes relating to immune function are differentially transcribed when fish are stressed by glochidia infestation than, for instance, by cadmium toxicity (Defo *et al*., 2019). Rates of stress-related gene transcription appear to differ greatly when infestation from different mussels is compared, indicating some degree of host-specific synchrony between host generalists and host specialists (Gendron *et al*., 2019).

Infestation can result in a darker colouration of wild-caught, wild-infested *S. trutta* when brought into the lab, indicating that the infested trout likely experienced higher general stress levels when acclimating to lab conditions than uninfested trout (Filipsson *et al*., 2016). No changes were noted in the mating colourations of wild-caught, lab-infested *Phoxinus phoxinus*, although the more highly infested hosts had lower fertility (as measured by sperm motility and sperm swimming curvature) and breeding tubercle number than individuals with low level of infestation (Kekäläinen *et al*., 2014). Furthermore, infestation has been shown to increase sensitivity to organic toxins such as crude oil and toluene, but not to alter bioaccumulation of cadmium (Moles, 1980; Defo *et al*., 2019).

## Behavioural response to glochidiosis

From the 14 studies on behavioural effects, 106 results were extracted (Table 2). This category had the highest proportion of grey literature with 5 non-peer-reviewed reports and should be viewed in that light. Given the high heterogeneity of response metrics and relatively few overall results, 5 broad categories were constructed: activity level, feeding, habitat use, migration and social interaction. The most well supported of the result categories were activity and feeding, with 9 and 6 studies, respectively. The same definition of results was used here as with those reported in the section on physiological responses of glochidiosis (statistical effect in text or figure, Supplementary Table S3).

**Table 2.** Reported results of specific behavioural effects experienced by host fishes during and shortly after glochidiosis

Category	Lower	No change	Higher	Papers represented	Mussel species represented	Fish species represented
Activity	11	32	7	9	5	4
Feeding	9	18	1	6	3	2
Habitat preference	3	13	2	4	2	2
Migration/migration direction	2	1	3	3	2	2
Social interaction	1	2	1	2	1	1

Further information as to the number of studies and species across which the results are described across are also reported.

Within the activity level category, 6 of the 9 studies investigated the *M. margaritifera*–*S. trutta* interaction (Table 2). General activity levels are often used in studies of animal behaviour, as it is relatively simple to measure, although the interpretation of the result can be highly dependent on context and individual variation (van Oers *et al*., 2005; Cha *et al*., 2012). The vast majority of the results in the activity level category (90%) were manipulative with the remaining 10% being non-manipulative.

In the feeding rate category, 5 of 6 studies investigated the *M. margaritifera*–*S. trutta* interaction, whereas 1 study investigated the divergent effect of 2 mussel species on 1 host species. Feeding rate is an ecologically relevant metric as differences in host feeding response can affect growth rates. Over half (60%) of the results used in this category were manipulative with the remaining 40% non-manipulative.

Collectively, the combinations of all other response categories had a similarly skewed species distribution as that of feeding rate, with 5 of the 8 studies investigating the *M. margaritifera*–*S. trutta* interaction, the other 3 investigating separate species interactions. This combination included studies on the impact of parasitism on social interactions, habitat preference and migration habits. Of the results from 28 studies, 82% were manipulative and 15% were non-manipulative with only 1 observational result.

### Activity levels

There is no clear indication that host activity levels are consistently significantly impacted the *M. margaritifera*–*S. trutta* interaction in the lab (Höglund, 2014; Filipsson *et al*., 2016; Gustavsson, 2019), although 2 correlations do show a negative relationship between activity and infestation rate (Taeubert and Geist, 2013; Filipsson *et al*., 2016). Two field studies demonstrate that parasitized individuals had lower average movement and dispersal tendency than controls, but when infested individuals did relocate, they relocated further (Freitt, 2016; Horký *et al*., 2019).

Both invasive (*S. woodiana*) and native (*Anodonta anatina*) species can have short-term effects on the activity levels of their host fishes, but the impact disappears later in the infestation (Horký *et al*., 2014; Slavík *et al*., 2017). *Cyprinus carpio* exhibited decreased movement at 4 dpi but not at 8 dpi (Slavík *et al*., 2017). *Squalius cephalus* had reduced overall activity levels at 4 and 7 dpi but not at 12 dpi or with a 30 day average. Moreover, no differences were observed in host diel behaviour or response to environmental variation (Horký *et al*., 2014).

Much like the case of gene response, a host may be affected differently by different mussels. *Etheostoma caeruleum* does not react in the same way to infestation from *P. occidentalis* as it does to infestation from *V. pleasii* (Crane *et al*., 2011). When infested with the former, fish moved less during feeding than the control group, a difference that magnified over 28 days of observation, but was unchanged when infested with *V. pleasii* at 14 dpi. When administered predator alarm ques, *V. pleasii*-infested individuals showed a non-significant trend towards higher feeding activity (Crane *et al*., 2011).

### Feeding

Infestation causes no short-term (⩽30 dpi) change in feeding tendencies in the *M. margaritifera*–*S. trutta* interaction, but does appear to have a greater negative impact at later infestation stages (⩽100 dpi) (Sunnerstam, 2013; Höglund, 2014; Österling *et al*., 2014; Filipsson *et al*., 2016; Gustavsson, 2019). Habitat complexity and interspecific competition have no effects on trout-feeding rates within the first 30 dpi (Sunnerstam, 2013; Höglund, 2014; Gustavsson, 2019). However, the relationship changes in the early infestation period (⩽100 dpi) as feeding behaviour has been negatively correlated with glochidial load and is occasionally observed as significantly reduced with respect to controls (Sunnerstam, 2013; Höglund, 2014; Österling *et al*., 2014; Filipsson *et al*., 2016). Furthermore, Höglund (2014) observed a higher food-spitting rate in infected trout than controls, possibly an effect of infested gills being inflamed and interfering with feeding (Thomas *et al*., 2014).

The feeding rate of *E. caeruleum* is generally unaffected by infestation from *P. occidentalis* and *V. pleasii*, but *V. pleasii*-infested individuals at 14 dpi consume more under predation risk stress than controls (Crane *et al*., 2011). While the literature on the impact of monoxenous parasites on foraging behaviour is mixed, Crane *et al*. (2011) suggest that fish parasitized with glochidia have decreased fitness as a function of increased energetic costs and therefore forage more to compensate for this (Milinski, 1985; Maksimowich and Mathis, 2000; Finley and Forrester, 2003; Crane *et al*., 2011; Krkošek *et al*., 2011).

### Habitat use, migration and social behaviour

Glochidiosis has been occasionally shown to cause minor differences in host habitat use, though 1 study (Horký *et al*., 2019) reports significant changes in habitat use as a function of behavioural thermoregulation (Horký *et al*., 2014; Freitt, 2016; Andersson, 2018; Horký *et al*., 2019). In the *M. margaritifera*–*S. trutta* interaction, no differences in use of water depth or velocity are reported compared to controls or glochidial load, with mixed reports on use of substrate size (Freitt, 2016; Andersson, 2018). *Squalius cephalus* infested with *A. anatina* were reported as spending more time at a greater distance from the bank, a difference not observed in the *M. margaritifera*–*S. trutta* interaction (Horký *et al*., 2014; Freitt, 2016). Behavioural thermoregulation (behavioural fever) is commonly observed in fish suffering from some infection or infestation, and is reported in the *M. margaritifera*–*S. trutta* interaction (Parker *et al*., 2011; Macnab and Barber, 2012; Boltaña *et al*., 2013; Mohammed *et al*., 2016; Horký *et al*., 2019).

The effects of infestation on migratory behaviour are mixed (Horký *et al*., 2014; Irmscher and Vaughn, 2015; Terui *et al*., 2017). A broad multi-host, multi-parasite re-capture study demonstrated that, in general, infested fishes dispersed upriver more than downriver (Irmscher and Vaughn, 2015). On the other hand, *S. cephalus* infested with *A. anatina* had a reduced upriver migratory tendency resultant from a preference to migrate at higher temperatures (Horký *et al*., 2014). One study on *Oncorhynchus masou masou* infested with *Margaritifera laevis* suggests a divergent effect induced by host size, with large fish relocating more and small fish less than uninfested individuals of the same sizes (Terui *et al*., 2017).

*Salmo trutta* infested with *M. margaritifera* shows no consistent difference in social behaviour than uninfested trout (Filipsson *et al*., 2017; Gustavsson, 2019). In habitats with low structural complexity, no correlation between the number of social interactions initiated and glochidial load was shown in a study with a non-manipulative design (Gustavsson, 2019) whereas a non-manipulative study described a negative correlation (Filipsson *et al*., 2016). In complex habitats, non-manipulated glochidial load was negatively related to the number of social interactions initiated (Gustavsson, 2019). The increase in habitat complexity offers visual barriers which therefore reduced overall interaction. The reduction in social interactions would allow the more heavily infested hosts to expend less energy, therefore improving their response to infestation (Gustavsson, 2019).

## Conclusions and future prospects

Parasitic mussels have low but significantly observable negative impacts on their host fish; the degree to which this impact is observable is highly dependent on the interspecific interaction, glochidial load and the time frame of interest (Kaiser, 2005; Chowdhury *et al*., 2019; Gendron *et al*., 2019; Marwaha *et al*., 2019; Horne, 2021). For example, long-term *M. margaritifera* infestation has mixed effects on salmonid host physiology, whereas the shorter infestation periods of other mussels are often negative (Douda *et al*., 2017; Marwaha *et al*., 2019). The metabolic and histopathological effects induced in the early infestation period definitively appear to have deleterious effects on hosts (Kaiser, 2005; Castrillo *et al*., 2019; Methling *et al*., 2019). With the exception of very high infestation loads, such effects, when present, generally disappear within a week (Kaiser, 2005; Crane *et al*., 2011; Castrillo *et al*., 2019; Horne, 2021). This phenomenon is likely caused by the combined effects of well-developed gill-wound healing and an increase in ventilation rates (Filipsson *et al*., 2017; Castrillo *et al*., 2019). Host behaviour generally appears unchanged. The changes observed in salmonid activity levels and feeding rates are likely the cause of the altered growth described earlier as the glochidia extracts minimal resources from the host (Österling *et al*., 2014; Denic *et al*., 2015; Filipsson *et al*., 2016; Horký *et al*., 2019). Evidence demonstrating that infested hosts tolerate stress differently than uninfested ones suggests that glochidiosis is a tolerable stressor at natural levels, but adds to the allosteric load of hosts when in combination with other stressors, at which point negative effects are observed (Dubansky *et al*., 2011; Filipsson *et al*., 2016; Gendron *et al*., 2019; Methling *et al*., 2019).

### Future prospects

The high skewness of species representation is a primary issue in research on glochidiosis, as comprehensive general-effect patterns can only be made in the *M. margaritifera*–*S. trutta* interaction, but the large number of reports stating no effect on growth and condition factor suggest a complex interaction is at play at natural infestation levels. General trends in the *M. margaritifera*–*S. trutta* interaction are difficult to bring to other mussel–fish interactions as the high host specificity, long infestation period and taxonomic distinction make *M. margaritifera* stand apart from most other mussels; a more generalizable mussel model should be found for the family Unionidae. A species from the genus *Anodonta* or *Unio* would be ideal given their worldwide distribution, which would allow for easily comparable intercontinental results. Enough evidence exists through a relatively small set of comparative papers to assert that not all hosts tolerate infestation from all mussels equally (Uthaiwan *et al*., 2003; Crane *et al*., 2011; Gendron *et al*., 2019). As such, particular care should be taken to compare the impacts of 1 mussel species on multiple hosts along with studies on the divergent impact of several mussel species on 1 host, as to better understand both the mode of action of this parasitic order as well as the factors behind host specificity.

Studies making claims based purely on non-manipulative results should be treated with care if no comparable finding is made evident by a similar manipulative result. As very few studies investigated the intraspecific physiological or immunological reasons for the differences in retained glochidial load (Reuling, 1919; Arey, 1923; Waller and Mitchell, 1989), efforts should be made to investigate and account for this. Future studies should measure aspects of host physiology, immunology and behaviour both before and after infestation (before after control impact) to compare not only the impact glochidia parasitism has on the host, but to also understand reasons for the differences in glochidia retention.

Research on [stress] coping style and the pace of life syndrome investigates the individual covariation of behavioural, immunologic and metabolic responses in relation to a given stressor (Koolhaas, 2008; Réale *et al*., 2010). As an example, coping styles have been associated with consistent inter-individual differences in metabolic rate, aggression (Øverli *et al*., 2004; Martins *et al*., 2011; Skov *et al*., 2019; Fu *et al*., 2021) and cortisol response (Koolhaas, 2008; Tudorache *et al*., 2015; Vargas *et al*., 2018; Wong *et al*., 2019), a hormone known to significantly impact glochidia retention (Kirk and Layzer, 1997; Dubansky *et al*., 2011). As such, correlations between glochidial load and activity levels or growth rates may not be causally related (Cunjak and McGladdery, 1991; Filipsson *et al*., 2016, 2017, Marwaha *et al*., 2019). While the literature surrounding coping style and pace of life syndrome research is far from conclusive (White *et al*., 2016; Krams *et al*., 2018; Royauté *et al*., 2018), evidence in its support offers a word of warning to conclusions drawn purely from non-manipulative results.

Many host-specific and parasite-specific factors such as fish species, age, size, condition, infestation history and mussel load are known to affect the rate of cyst formation. However, observations showing differential cyst growth on closely attached glochidia indicate that some signal compound excreted by the glochidia likely influences the exact tissue response (Nezlin *et al*., 1994; Rogers-Lowery and Dimock, 2006). The presence of such a compound may be given further evidence from results demonstrating altered liver functionality following infestation (Douda *et al*., 2017; Slavík *et al*., 2017). Different modes of action are clearly employed by different mussels (generalists *vs* specialists), however, for both mussel strategy types, high initial larval rejection is not uncommon (Crane *et al*., 2011; Gendron *et al*., 2019). As parasitic mussels often live longer than their hosts, fish have more opportunities to advance their anti-mussel defences in the co-evolutionary arms race. One might speculate that, to compensate for this disadvantage, mussels may produce highly variable glochidia thereby allowing them to account for many possible variations of host-defence mechanisms. Bauer (1994) offers a similar suggestion when detailing the differences in size of glochidia and mussel fertility between the specialized Margaritiferidae mussels and the more generalist *Anodonta* species. This evolutionary strategy would reduce the instantaneous infestation success rate but improve continued infestation potential by anticipating future adaptation in host-defence capabilities. Such a strategy would be analogous to the trade-offs between generalist and specialist phenotypic strategies (Haaland *et al*., 2020). A better understanding of the molecular factors affecting cyst formation, particularly those of glochidial origin, will better inform how immunity functions.

Of the almost 200 individual results from the molecular effects category within the physiological effects section, half (Supplementary Table S2; 89/191) come from 1 study of questionable usefulness (low sample size and lack of direct controls, Uthaiwan *et al*., 2003). As such the low number of studies on the transcriptomic and molecular changes (12 combined) induced by infestation should be addressed, particularly given the broad nature of this result category. Offering a more focused measure of host effects and stress than whole-body effects on their own, this area of research allows for a detailed proximate level explanation as to the exact nature of host tolerance of parasitism, in exchange for lower ecological relevance. For example, both Douda *et al*. (2017) and Slavík *et al*. (2017) show a decrease in liver functionality following infestation, while providing valuable insight into the metabolomics associated with glochidia parasitism it is of less ecological relevance than impacts on foraging behaviour. Likewise, transcriptomic work offers crucial information as to why there is such variation in observed effects, but is not of direct ecological relevance (Gendron *et al*., 2019).

Infestation intensity appears to play a significant role in determining if infestation will be of significant impact on the host. This is worthy of note when comparing results by Chowdhury *et al*. (2019) and Marwaha *et al*. (2019) on 1+ *S. trutta* infested with high and low levels of *M. margaritifera*, respectively. While the higher infestation level reduced growth, the lower infestation level resulted in hosts with higher condition factors than controls. This is an important result for both theoretical parasitic ecology and future mussel conservation efforts. PITT suggests that heteroxenous parasites will cause changes likely to increase the risk of predation for the host. In the case of a monoxenous parasite, one would expect to see reduced trophic facilitation as evidenced by Marwaha *et al*. (2019). Improvements in condition and survival of fish in response to other parasitic infestations have been previously reported (Milinski, 1985; Arnott *et al*., 2000; Museth, 2001; Loot *et al*., 2002; Ondračková *et al*., 2004). One might postulate that mussels with shorter encystment periods have a reduced risk of predation than long-infesting mussels (as a function of infestation length). These short-term mussels may take advantage of this reduced risk factor by putting more stress on the host through a higher nutrient extraction rate per unit time, ultimately causing greater harm to the host than long-infesting mussels. This is evidenced by a higher proportion of negative effects to no effects being reported with *M. margaritifera* than all other mussels (Supplementary Tables S2 and S3). Future research into the divergent impact of mussels with long and short infestation lengths may shed light on this hypothesis. The literature on migration and relocation behaviours should also be broadened to investigate this effect, as longer infestation periods would, theoretically, increase mussel dispersal. If the beneficial effects of low-level infestation can be supported more thoroughly this could vastly improve the success of mussel reintroduction and conservation efforts.

Conservation efforts for unionid mussels typically fall into 2 forms: the first involves allowing hosts to naturally spread the metamorphosed larva across the conservation area, while the second collects the metamorphosed larvae *in vitro* for manual dispersal. The first method is both fast and inexpensive as the time frame from animal collection to re-release usually takes less than a month and can be done with minimal equipment and investment. A common practice is to place both fish and mussels in enclosures directly in the field, allowing for a semi-natural infestation with excess glochidia being directly released into the wild. The second is more expensive, generally requiring a dedicated facility and specialized equipment (Thomas *et al*., 2010; Donaldson *et al*., 2019). Moreover, the second method takes longer than the minimum infestation length of the mussel in question, a factor that can add significant expenses to the practice as maintaining a large number of fish for almost a year (in the case of *M. margaritifera* conservation particularly) is not inexpensive.

In this light, the second conservation method may be the most appropriate for mussels with shorter infestation times. Not only does the shorter infestation time result in lower maintenance costs, but the negative effects induced by glochidiosis can be mitigated and corrected for in captivity; a noteworthy benefit as evidence gathered here suggests that glochidiosis from short-infesting mussels induces more negative effects than long-infesting mussels. On the other hand, for *M. margaritifera*, the first conservation method may be the most appropriate, as lower infestation loads could increase long-term host survival. However, given that the first conservation method is commonly used for both infestation lengths, holding the hosts for a longer period post-infestation would ensure full recovery prior to release into the wild.

Taken together the current literature on the impacts of glochidia infestation is, while sparse, quite broad. Enough basal research has been published to allow for nuanced questions around the complex interactions between parasitic mussels and host fishes to be asked in future studies, although the literature on non-Margaritiferidae mussels requires significant investment to account for the higher diversity and worldwide distribution. Future molecular and genetic studies on the divergent impacts of multiple mussel species on a host, as well as the differential tolerance of multiple fish species to a single parasite, would provide valuable insight into the mode of action of this unique mollusc order, and provide a better understanding of how immunity to the parasite works and is formed. Further studies on the behavioural effects of infestation, particularly activity and migration, will better inform the causes of the observed physiological impacts as well as assist in explaining and categorizing the genetic mosaic of mussel populations for preservation efforts. Furthermore, the conclusions from this review offer clear ways to improve reintroduction practices with no significant increases to overall costs.

## Supplementary material

For supplementary material accompanying this paper visit https://doi.org/10.1017/S0031182022001226. click here to view supplementary material
