# Supplementary material for: Effects of parasitic freshwater mussels on their host fishes: a review
Source: Parasitology. 2022 Sep 2;149(14):1958–75. doi: 10.1017/S0031182022001226 (PMC10090606; doi:10.1017/S0031182022001226)
Supplement: Supplementary file 1 [file S0031182022001226sup.zip › S0031182022001226sup001.docx]

**Supplementary Table S1**

| 1st Author | Date | Title | Mussle Species | Host Species | Origin | Study focus |
| --- | --- | --- | --- | --- | --- | --- |
| Andersson | 2018 | The effect of the freshwater pearl mussel (*Margaritifera margaritifera*) on growth, condition factor and habitat choice of brown trout (*Salmo trutta*). (English) | *Margaritifera margaritifera* | *Salmo trutta* | Karlstad | growth / condition actor / behavior |
| Arey | 1923 | Observations on an acquired immunity to a metazoan parasite | *Lampsilis luteola* | *Micropterus Salmoide* | In Dodd 2006 | acquired immunity |
| Arey | 1932a | The nutrition of glochidia during metamorphosis. A microscopical study of the sources and manner of utilization of the nutritive substances | *Lampilis luteola / Anodonta corpulenta / Hemilastena ambigua* | *Micropterus Salmoides / Lepomis humilis / Necturus maculatus* | Cited Arey 1924 | nutrient transfer |
| Arey | 1932b | The formation and structure of the glochidial cyst | *Lampsilis luteola / Hemilastena ambigua / Anodonta corpulent / Lampsilis anodontoides* | *Micropterus Salmoides / Lepisosteus osseus /* urodele *Necturus / Lepomis humilis* | In Hastie 2001 | histopathology |
| Arey | 1932c | A microscopical study of glochidial immunity | *Lampsilis anoclontoides / Lampsilis lutcola* | gar pikes */ Micropterus Salmoides* | In Rogers 2003 | acquired immunity |
| Bruno | 1988 | Natural infection of farmed Atlantic Salmon, *Salmo sarar* L., parr by glochidia of the freshwater pearl mussel, *Margaritifera margaritifera* L. | *Margaritifera margaritifera* | *Salmo salar* | In Treasurer 2006 | growth |
| Castrillo | 2019 | Early stages of *Margaritifera margaritifera* glochidiosis in Atlantic Salmon: Morphopathological characterization | *Margaritifera margaritifera* | *Salmo salar* | WOS | histopathology |
| Chowdhury | 2017 | Effect of Freshwater Pearl Mussel (*Margaritifera margaritifera*) infection on virulence of *Flavobacterium columnare* in Brown trout (*Salmo trutta*) | *Margaritifera margaritifera* | *Salmo trutta* | GOS | coinfection |
| Chowdhury | 2018 | Interaction between the endangered freshwater pearl mussel *Margaritifera margaritifera*, the duck mussel *Anodonta anatina* and the fish host (Salmo) : acquired and cross-immunity | *Margaritifera margaritifera / Anodonta anatina* | *Salmo trutta* | Cites Hastie 2001 | acquired immunity |
| Chowdhury | 2019 | Effect of glochidia infection on growth of fish: freshwater pearl mussel *Margaritifera margaritifera* and brown trout *Salmo trutta* | *Margaritifera margaritifera* | *Salmo salar / Salmo trutta* | GOS | weight |
| Crane | 2011 | Do gill parasites influence the foraging and antipredator behaviour of rainbow darters, *Etheostoma caeruleum*? | *Ptychobranchus occidentalis / Venustaconcha pleasii* | *Etheostoma caeruleum* | WOS | behavior |
| Cunjak | 1991 | The parasite-hote relationship of glochidia (Molusca, Margaritiferidea) on the gills of young-of-the-year Atlantic salmon (*Salmo salar*) | *Margaritifera margaritifera* | *Salmo salar* | WOS | condition factor |
| Defo | 2019 | Cumulative effects of cadmium and natural stressors (temperature and parasite infection) on molecular and biochemical responses of juvenile rainbow trout | *Strophitus undulatus* | *Oncorhynchus mykiss* | Cites Rogers-Lowery 2006 | toxicity / gene transcription |
| Denic | 2015 | Trophic relationships between the larvae of two freshwater mussels and their fish hosts | *Margaritifera margaritifera / Unio crassus* | *Phoxinus phoxinus / Salmo trutta* | cited Taeubert 2013 | nutrient transfer |
| Dodd | 2006 | Persistence of host response against glochidia larvae in *Micropterus salmoides* | *Lampsilis reeveiana* | *Micropterus salmoides* | GOS | acquired immunity |
| Donrovich | 2017 | Invasive Chinese pond mussel *Sinanodonta woodiana* threatens native mussel reproduction by inducing cross-resistance of host fish | *Sinanodonta woodiana / Anodonta anatina* | *Squalius cephalus* | WOS | acquired immunity |
| Douda | 2017 | Direct impact of invasive bivalve (*Sinanodonta woodiana*) parasitism on freshwater fish physiology: evidence and implications | *Sinanodonta woodiana* | *Squalius cephalus* | WOS | weight / condition factor / hematology / enzyme activity / |
| Du | 2015 | Effect of infection stress by glochidia of *Hyriopsis* *cumingii* on respiratory metabolism in yellow catfish, *Pelteobagrus fulvidraco* | *Hyropsis cumingii* | *pelteobargrus fulvidraco* | Ma 2018 | oxygen usage/ ammonia excretion / mortality |
| Dudansky | 2011 | Influence of Cortisol on the Attachment and Metamorphosis of Larval *Utterbackia imbecillis* on Bluegill Sunfish (*Lepomis macrochirus*) | *Utterbackia imbecillis* | *Lepomis macrochirus* | GOS | stress |
| Filipsson | 2016 | Heavy loads of parasitic freshwater pearl mussel (*Margaritifera margaritifera* L.) larvae impair foraging, activity and dominance performance in juvenile brown trout (*Salmo trutta* L.) | *Margaritifera margaritifera* | *Salmo trutta* | WOS | behavior / coloration |
| Filipsson | 2017 | Encystment of parasitic freshwater pearl mussel (*Margaritifera margaritifera*) larvae coincides with increased metabolic rate and haematocrit in juvenile brown trout (*Salmo trutta*) | *Margaritifera margaritifera* | *Salmo trutta* | WOS | metabolic rate / hematology |
| Freitt | 2016 | The effect of the freshwater pearl mussel (*Margaritifera margaritifera*) on movement, habitat choice, and growth of brown trout (*Salmo trutta*) | *Margaritifera margaritifera* | *Salmo trutta* | Karlstad | behavior / habitat choice / growth |
| Fritts | 2013 | Shifts in stable-isotope signatures confirm parasitic relationship of freshwater mussel glochidia attached to host fish | *Lampsilis cardium* | *Micropterus Salmoides* | In Horky 2014 | nutrient transfer |
| Fustish | 1978 | Glochidiosis of Salmonid Fishes. II. Comparison of Tissue Response of Coho and Chinook Salmon to Experimental Infection with *Margaritifera margaritifera* (L.) (Pelecypoda: Margaritanidae) | *Margaritifera margaritifera* | *Oncorhynchus kisutch / Oncorhynchus tshawytsch* | Cites Karna 1973 | histopathology |
| Gendron | 2019 | Stress-related gene transcription in fish exposed to parasitic larvae of two freshwater mussels with divergent infection strategies | *Elliptio complanata / Lampsilis radiata* | *Perca flavescens* | GOS | gene transcription |
| Gopko | 2018 | Interactions between two parasites of brown trout (*Salmo trutta*): Consequences of preinfection | *Margaritifera margaritifera* | *Salmo trutta* | WOS | coinfection |
| Gustavsson | 2019 | The effects of habitat complexity and infections from freshwater pearl mussel larvae on brown trout interactions (swedish) | *Margaritifera margaritifera* | *Salmo trutta* | Karlstad | behavior |
| Hanrahan | 2019 | Field and laboratory investigations of *Echyridella menziesii* (Unionida: Hyriidae) interactions with host fishes | *Echyridella menziesii* | *Gobiomorphus cotidianus* | Cites Nezlin 1994 | acquired immunity |
| Hoglund | 2014 | The freshwater pearl mussel´s effect on the trout´s foraging and prey handling over time (Swedish) | *Margaritifera margaritifera* | *Salmo trutta* | Karlstad | behavior |
| Horky | 2014 | Parasite-induced alterations of host behaviour in a riverine fish: the effects of glochidia on host dispersal | *Anodonta anatina* | *Squalius cephalus* | WOS | behavior |
| Horky | 2019 | Altered thermoregulation as a driver of host behaviour in glochidia-parasitised fish | *Margaritifera margaritifera* | *Salmo trutta* | WOS | behavior |
| Horne | 2021 | The effects of glochidia infestation on the metabolic rate and hypoxia tolerance of bluegill *Lepomis macrochirus* and largemouth bass *Micropterus salmoides* | *Lampsilis straminea* | *Lepomis macrochirus / Micropterus salmoides* | GOS | metabolic rate |
| Howerth | 2006 | Experimentally induced glochidiosis in smallmouth bass (*Micropterus dolomieu*) | *Actinonaias pectorosa* | *Micropterus dolomieu* | WOS | histopathology |
| Irmscher | 2015 | Limited movement of freshwater mussel fish hosts in a southern US river | *Many* | *Many* | WOS | behavior |
| Kaiser | 2005 | The effects of glochidiosis on fish respiration | *Mapsilis reeveiana* | *Micropterus Salmoides* | In Crane 2011 | metabolic rate |
| Karna | 1978 | Glochidiosis of Salmonid fishes. III. Comparative susceptibility of natural infection with *Margaritifera margaritifera* (L.) (Pelecypoda: Margaritanidae) and associated histopathology. | *Margaritifera margaritifera* | *Oncorhynchus tshawytsch / Oncorhynchus kisutch / Salmo clarki / Salmo gairdneri* | in Treasurer 2000 | histopathology |
| Kekalainen | 2014 | Do highly ornamented and less parasitized males have high quality sperm? - an experimental test for parasite-induced reproductive trade-offs in European minnow (*Phoxinus phoxinus*) | *Anodonta anatina* | *Phoxinus phoxinus* | WOS | sperm quality / coloration |
| Ma | 2018 | Effects of infection stress by glochidia of freshwater mussel *Hyropsis cumingii* on main nutritional indicies in yellow catfish *Pelteobargrus fulvidraco* (chinese) | *Hyropsis cumingii* | *Pelteobargrus fulvidraco* | Dodd 2006 | biochemical indices |
| Marwaha | 2019 | Host (*Salmo trutta*) age influences resistance to infestation by freshwater pearl mussel (*Margaritifera margaritifera*) glochidia | *Margaritifera margaritifera* | *Salmo trutta* | WOS | hematology / condition factor |
| Methling | 2018 | Energetic costs in the relationship between bitterling and mussels in East Asia | *Sinanodonta woodiana* | *Rhodeus ocellatus* | WOS | metabolic rate / |
| Methling | 2019 | Intensity-dependent energetic costs in a reciprocal parasitic relationship | *Sinanodonta woodiana* | *Rhodeus amarus* | WOS | metabolic rate |
| Meyers | 1980 | Glochidiosis of Salmonio Fishes. IV. Humoral and Tissue Responses of Coho and Chinook Salmon to Experimental Infection with *Margaritifera margaritifera* (L.) (Pelecypoda: Margaritanidae) | *Margaritifera margaritifera* | *Oncorhynchus kisutch / Oncorhynchus tshawytsch* | Cites Karna 1973 | Immunology / histopathology |
| Moles | 1980 | Sensitivity of Parasitized Coho Salmon Fry to Crude Oil, Toluene, and Naphthalene | *Anodonta oregonen* | *Oncorhynchus kiutch* | In Moles 1983 | toxicology |
| Moles | 1983 | Effect of Parasitism by Mussel Glochidia on Growth of Coho Salmon | *Anodonta oregonensis* | *Oncorhynchus kisutch* | WOS | growth |
| Murphy | 1942 | Relationship of the freshwater mussel to trout in the Truckee River | *margaritifera margatitifera* | *Oncorhynchus mykiss* | In Karna 1973 | - |
| Nezlin | 1994 | Glochidium morphology of the freshwater pearl mussel (*Margaritifera margaritifera*) and glochidiosis of Atlantic salmon (*Salmo salar*) - a study by scanning electron-microscopy | *Margaritifera margaritifera* | *Salmo salar* | WOS | histopathology / mortality |
| O'Connel | 1999 | Evidence of immunological Responses by a Host Fish (*Ambloplites rupestris*) and Two Non-Host Fishes (*Cyprinus carpio* and *Carassius auratus*) to Glochidia of a Freshwater Mussel (*Villosa iris*) | *Villosa iris* | *Ambloplites rupestris / Cyprinus carpio / Carassius auratus* | In Rogers 2003 | immunology |
| Ooue | 2017 | A delayed effect of the aquatic parasite *Margaritifera laevis* on the growth of the Salmonid host fish *Oncorhynchus masou masou* | *Margaritifera laevis* | *Oncorhynchus masou masou* | WOS | growth |
| Osterling | 2014 | Parasitic freshwater pearl mussel larvae (*Margaritifera margaritifera* L.) reduce the drift-feeding rate of juvenile brown trout (*Salmo trutta* L.) | *Margaritifera margaritifera* | *Salmo trutta* | WOS | behavior |
| Reuling | 1919 | Acquired immunity to an animal parasite | *Lampsilis anodontoides* | *Lepisosteus osseus / Lepisosteus platostomus* | In Rogers 2003 | acquired immunity |
| Rogers | 2003 | Acquired resistance of bluegill sunfish *Lepomis macrochirus* to glochidia larvae of the freshwater mussel *Utterbackia imbecillis* (Bivalvia : Unionidae) after multiple infections | *Utterbackia imbecillis* | *Lepomis macrochirus* | WOS | acquired immunity |
| Rogers-Lowery | 2006 | Encapsulation of attached ectoparasitic glochidia larvae of freshwater mussels by epithelial tissue on fins of naive and resistant host fish | *Utterbackia imbecillis* | *Lepomis macrochirus* | WOS | histopathology |
| Rogers-Lowery | 2007 | Antibody response of bluegill sunfish during development of acquired resistance against the larvae of the freshwater mussel *Utterbackia imbecillis* | *Utterbackia imbecillis* | *Lepomis macrochirus* | cites Reuling 1919 | acquired immunity |
| Seshaiya | 1969 | Some observations on the life-histories of south Indian freshwater mussels | *Lamellidens spp.* | *Ophiocephalus spp.* | Arey 1923a | acquired resistance |
| Silva-Souza | 2002 | The Histopathology of the Infection of *Tilapia rendalli* and *Hypostomus regani* (Osteichthyes) by Lasidium Larvae of *Anodontites trapesialis* (Mollusca, Bivalvia) | *Anodontites trapesialis* | *Tilapia rendalli / Hypostomus regani* | Cites Meyers 1977 | histopathology |
| Slavik | 2017 | Parasite-induced increases in the energy costs of movement of host freshwater fish | *Sinanodonta woodiana* | *Cyprinus carpio* | GOS | metabolic rate / behavior / hematology |
| Sunnerstam | 2013 | Vilken inverkan har flodpärlmusslans (*Margaritifera margaritifera*) glochidielarver på öringens (*Salmo trutta*) födointag vid olika tidpunkter? (Swedish) | *Margaritifera margaritifera* | *Salmo trutta* | Karlstad | behavior |
| Taeubert | 2013 | Critical swimming speed of brown trout (*Salmo trutta*) infested with freshwater pearl mussel (*Margaritifera margaritifera*) glochidia and implications for artificial breeding of an endangered mussel species | *Margaritifera margaritifera* | *Salmo trutta* | WOS | behavior |
| Terui | 2017 | Parasite infection induces size-dependent host dispersal: consequences for parasite persistence | *Margaritifera laevis* | *Oncorhynchus masou masou* | Cites Hastie 2001 | behavior |
| Thomas | 2014 | Does the parasitic freshwater pearl mussel *M. margaritifera* harm its host? | *Margaritifera margaritifera* | *Salmo trutta* | WOS | spleen / histopathology / hematology / respiration |
| Treasurer | 2000 | The pathology and seawater performance of farmed Atlantic Salmon infected with glochidia of *Margaritifera margaritifera*. | *Margaritifera margaritifera* | *Salmo salar* | In Dodd 2006 | histopathology / plasma chloride / mortality / weight |
| Treasurer | 2006 | Effects of (*Margaritifera margaritifera*) glochidial infection on performance of tank-reared Atlantic Salmon (*Salmo salar*) | *Margaritifera margaritifera* | *Salmo salar* | WOS | stress / growth / condition factor / immunity |
| Uthaiwan | 2003 | Studies on the plasma composition of fish hosts of the freshwater mussel, *Hyriopsis myersiana*, with implications for improvement of the medium for culture of glochidia | *Hyriopsis myersiana* | *Cyprinus carprio / Oreochromis nifotica / (Clarias macrocephalus x C. gariepinus / Pangasius pangmius* | WOS | hematology |
| Waller | 1989 | Gill tissue reactions in walleye *Stizostedion vitreum vitreum* and common carp *Cyprinus carpio* to glochidia of the freshwater mussel *Lampsilis radiata siliquoidea* | *Lampsilis radiata siliquoidea* | *Stizostedion vitreum vitreum / Cyprinus carpio* | In Rogers 2003 | histopathology |
| Watters | 1996 | Shedding of Untransformed Glochidia by Fishes Parasitized by *Lampsilis fasciola* Rafinesque, 1820 (Mollusca: Bivalvia: Unionidae): Evidence of Acquired Immunity in the Field? | *Lampsilis fasciola* | many | Cites Fustish 1978 | acquired immunity |
| Wen | 2009 | The Host Efficiency of Two Kinds of Fish for Glochidia of *Hyriopsis cumingii* and Impact on Plasma Biochemical Indexes of Host Fish | *Hyriopsis cumingii* | *Pelteobagrus fulvidraco / Tilapia nilotica* | GOS | hematology |
| Young | 1987 | Infection and successful reinfection of brown trout (*Salmo trutta*(L.)) with glochidia of *Margaritifera margaritifera*(L.). | *Margaritifera margaritifera* | *Salmo trutta* | In Cunjak 1991 | acquired immunity |
| Ziuganov | 2005 | A paradox of parasite prolonging the life of its host. Pearl mussel can disable the accelerated senescence program in Salmon | *Margaritifera margaritifera* | *Salmo salar* | WOS | hormonal / mortality |
| Zou | 2017 | Formation of parasitic cysts of *Hyriopsis cumingii* larvae on the gill filaments of three kinds of fish | *Hyriopsis cumingii* | *Pelteobagrus fulvidraco / Cyprinus carpio / P. fulvidraco* infested again | In Ma 2018 | histopathology / immunity |
