# Supplementary material for: Effects of parasitic freshwater mussels on their host fishes: a review
Source: Parasitology. 2022 Sep 2;149(14):1958–75. doi: 10.1017/S0031182022001226 (PMC10090606; doi:10.1017/S0031182022001226)
Supplement: Supplementary file 1 [file S0031182022001226sup.zip › S0031182022001226sup002.docx]

**Supplementary Table S2**

| Legend | |
| --- | --- |
| * | Manipulative study |
| ¤ | Non-manipulative study |
| ~ | Value not specified in paper, circumstantial information provided in paper informs an estimated value. Accuracy of estimations vary. |
| gl/f | glochidia per fish |
| gl/fg | glochidia per gram of fish |
| dpi | days post infestation |
| (W) | Wild caught fish |
| (H) | Hatchery reared fish |

| Mussel species | Fish species | Specific effect | Glochidia load | Against | DPI | + -0 | p | Correlation Coefficient | n= | Paper |
| --- | --- | --- | --- | --- | --- | --- | --- | --- | --- | --- |
| Whole body effects | | | | | | | | | | |
| *Margaritifera margaritifera* | *Salmo trutta* 0+ (W) | Growth during infestation period | / | Control * | 30 - 33 dpi | - | 0,046 | / | 159 | Andersson, 2018 |
|  |  | Condition Factor (k) during infestation period |  |  |  | 0 | 0,727 |  |  |  |
|  |  | Growth in the field |  |  | 65 - 75 dpi | 0 | 0,17 |  | 62 |  |
|  |  | Condition Factor (k) in the field |  |  |  | 0 | 0,207 |  |  |  |
|  | *Salmo trutta* 0+ (H) | Condition factor (k) | 4.5 gl/f | Glochidia Load ¤ | 60 dpi | 0 | 0,887 | 0,0186 | 30 | Marwaha et la, 2019 |
|  |  | Weight |  |  |  | 0 | 0,857 | -0,0238 |  |  |
|  |  | Length |  |  |  | 0 | 0,334 | -0,127 |  |  |
|  |  | Condition factor (k) |  | Glochidia Load ¤ | 200 dpi | 0 | 0,481 | -0,1337 |  |  |
|  |  | Weight |  |  |  | 0 | 0,956 | -0,01 |  |  |
|  |  | Length |  |  |  | 0 | 0,7531 | 0,0599 |  |  |
|  |  | Condition factor (k) |  | Glochidia Load ¤ | 300 dpi | - | 0,0817 | -0,2051 |  |  |
|  |  | Weight |  |  |  | - | 0,0006 | -0,288 |  |  |
|  |  | Length |  |  |  | - | 0,003 | -0,256 |  |  |
|  |  | Condition factor (k) |  | Control * | 300 dpi | 0 | 0,865 | / | 70 |  |
|  |  | Weight |  |  |  | 0 | 0,514 |  |  |  |
|  |  | Length |  |  |  | 0 | 0,232 |  |  |  |
|  | *Salmo trutta* (W) | Fork Length | 1 to 309 gl/f | Control ¤ | ~50 dpi | 0 | >0.1 | / | 28 | Filipsson et al, 2016 |
|  |  | Weight |  |  |  | 0 | >0.1 |  |  |  |
|  | *Salmo trutta* 1+ (H) | Growth | 7889 ± 390 gl/f or 138 ± 3 gl/fg | Control * | 84 dpi | - | <0.094 | / | ~319 | Chowdhury et al, 2019 |
|  |  | Survival |  |  |  | 0 | / |  |  |  |
|  |  | Growth | 5473 ± 445 gl/f | Control * | 203 dpi | - | <0.003 | / |  |  |
|  |  | Survival |  |  |  | 0 | / |  |  |  |
|  |  | Growth | 5202 ± 804 gl/f | Control * | 266 dpi | - | <0.002 | / |  |  |
|  |  | Survival |  |  |  | 0 | / |  |  |  |
|  |  | Growth | 3125 ± 566 gl/f | Control * | 315 dpi | - | <0.006 | / |  |  |
|  |  | Survival |  |  |  | 0 | / |  |  |  |
|  | *Salmo trutta* 1+ (W) | Fork Length | Between 0 and 11 gl/fg or ~66 gl/f | Control ¤ | ~250 dpi | 0 | >0.45 | / | 62 | Filipsson et al, 2017 |
|  |  | Weight |  |  |  | 0 | >0.45 |  |  |  |
|  |  | Condition factor (k) |  |  |  | 0 | >0.45 |  |  |  |
|  | *Salmo trutta* 1+ (H) | Condition factor (k) | 212.8 gl/f Infestation intensity category cutoff at 200 gl/f | Glochidia Load ¤ | 300 dpi | + | 0,0368 | 0,3054 | 50 | Marwaha et la, 2019 |
|  |  |  |  | High vs low infestation ¤ |  | + | 2.73e−07 | / |  |  |
|  |  |  |  | Control * |  | + | 0,0001 |  |  |  |
|  |  | Weight |  | High glochidia load (>200) ¤ |  | 0 | 0,06 | 0,3968 |  |  |
|  |  | Length |  |  |  | 0 | 0,055 | 0,4052 |  |  |
|  | *Salmo salar* 0+ (H) | Growth in freshwater | ~282 gl/f | Control ¤ | ~150 dpi | 0 | / | / | ~24 | Bruno et al, 1988 |
|  |  | Survival in freshwater |  |  |  | 0 |  |  |  |  |
|  |  | Growth in saltwater | ~116 gl/f | Control ¤ | ~250 dpi | 0 | / | / | ~29 |  |
|  |  | Survival in saltwater |  |  |  | 0 |  |  |  |  |
|  | *Salmo salar* 0+ (W) | Fork Length | pooled avg of 191 gl/f at one site and 31 gl/f | Glochidia Load ¤ | ~250 dpi | 0 | >0.6 & >0.3 | 0.065 & -0.117 | ~74 | Cunjak & McGladdery, 1991 |
|  |  | condition factor (k) of all glochidia loads |  |  |  | 0 | <0.005 | -0,24 |  |  |
|  |  | condition factor (k) for glochidia load >200 | >200 gl/f |  |  | - | <0.005 | -0,56 | 24 |  |
|  |  | condition factor (k) | pooled avg of 191 gl/f at one site and 31 gl/f | Between field sites ¤ | ~250 dpi | 0 | >0.2 | / | ~74 |  |
|  |  | Survival |  |  |  | - | / |  |  |  |
|  | *Salmo salar* (H) | Length | 818 ± 377 gl/f at 14dpi 1392 ± 641 gl/f at 42dpi 1385 ± 553 gl/f @70dpi ~81± 121 gl/f at ~105dpi <1 ± 2.3 gl/f at ~182 dpi | Control * | ~30 dpi | 0 | 0,51 | / | 600 | Treasurer et al, 2006 |
|  |  | Weight |  |  |  | 0 | 0,64 |  |  |  |
|  |  | Length |  |  | ~105 dpi | - | 0,0006 |  |  |  |
|  |  | Weight |  |  |  | - | 3E-05 |  |  |  |
|  |  | Length |  |  | ~182 dpi | - | 0,0004 |  |  |  |
|  |  | Weight |  |  |  | 0 | / |  |  |  |
|  |  | Length |  |  | ~273 dpi | 0 | 0,1172 |  |  |  |
|  |  | Weight |  |  |  | 0 | 0,13 |  |  |  |
|  |  | Condition factor (k) |  |  |  | 0 | >0.05 |  |  |  |
|  |  | Survival |  |  |  | 0 | / |  |  |  |
|  | *Salmo trutta* (W) | Specific Growth rate | High infestation > 226 ± 24 Low infestation <18±15 | Low infestation ¤ | ~100 dpi | - | 0,003 | / | 21 | Freitt 2016 |
|  |  | Specific growth rate (only 1+) |  |  |  | - | 0,004 |  | / |  |
|  |  | Initial condition factor |  |  | ~15 dpi | 0 | >0.05 |  | 18 |  |
|  |  | secondary condition factor |  |  | ~100 dpi | 0 |  |  | 21 |  |
|  |  | Change in condition factor |  |  |  | 0 |  |  | / |  |
|  | *Salmo salar* >1+ (W) | Post-spawning survival | 2000 to 7000 gl/f | Observation ¤ | / | + | / | / | / | Zuiganov et al, 2005 |
|  |  | Survival after temporary asphyxia | 65 to 400+ gl/f | Control * | / | + | < 0.05 | / | / |  |
|  |  | Survival after gill burn |  |  |  | + |  |  |  |  |
|  |  | Survival after hook wounds |  |  |  | + |  |  |  |  |
|  | *Salmo salar* 1+ (H) | Survival | 66 to 219 gl/f (up to 1000 in some cases) | Control * | ~438 dpi | 0 | >0.05 | / | 100 | Treasurer & Turnbull 2000 |
|  |  | Weight |  |  |  | 0 | >0.05 |  |  |  |
|  |  | Specific growth rate |  |  |  | 0 | >0.05 |  |  |  |
| *Margaritifera margaritifera falcata* | *Salmo trutta* 0+ (H) | Survival | 100-295 gl/f | Observation ¤ | ~59 dpi | - | / | / | 200 | Murphy, 1942 |
|  | *Oncorhynchus mykiss* 0+ (H) | Survival | 600-1200 gl/f |  | 1 dpi | - | / | / | 400 |  |
|  |  | Survival | 250-750 gl/f |  | ~59 dpi | - | / | / | 400 |  |
|  |  | Survival | 30 - 50 gl/f |  | ~23 dpi | 0 | / | / | 100 |  |
| *Margaritifera laevis* | *Oncorhynchus masou masou* 0+ (H) | Growth rate | 48 ± 29 gl/f | Control * | 50 dpi | 0 | / | / | 75 | Ooue, et al, 2017 |
|  |  |  |  |  | 70 dpi | - |  |  |  |  |
| *Strophitus undulatus* | *Oncorhynchus mykiss* (H) | Condition factor (K) | 25.46 ± 2.64 gl/f on whole body 7.69 ± 2 on gills | Control * | 28 dpi | 0 | >0.05 | / | 9 | Defo et al, 2019 |
| Anodonta oregonen | *Oncorhynchus kisutch* 0+ (H) | Weight | 0 to 50 gl/f. 50+ gl/f dies within 30 days | Glochidia load ¤ | 90 dpi | - | < 0.05 | / | 2200 | Moles 1983 |
|  |  | Survival |  |  |  | - | / | / |  |  |
| *Anodonta anatina* | *Rhodeus amarus* (W) | Weight | 19 gl/f on whole body & 2 gl/f on gills | Control * | 4 dpi | 0 | 0,241 | / | 48 | Methling et al, 2019 |
| *Sinanodonta woodiana* | *Squalius cephalus* 1+ (H) | Weight | 120 ± 39 gl/f | Control * | 43 dp first infestation, 3 dp last infestation | - | >0.001 | / | 16 | Douda et al, 2017 |
|  |  | Length |  |  |  | 0 | >0.05 |  |  |  |
|  |  | Condition factor (k) |  |  |  | - | >0.001 |  |  |  |
|  |  | Weight | 25 ± 4 gl/f | Control * |  | 0 | <0.001 | / | 16 |  |
|  |  | Length |  |  |  | 0 | >0.05 |  |  |  |
|  |  | Condition factor (k) |  |  |  | - | 0,08 |  |  |  |
| *Hyriopsis cumingii* | *Pelteobagrus fulvidraco* | Survival | 2480 ± 114 gl/f in low group and 3244 ± 248 gl/f in high group | Glochidia Load * | 12 dpi | - | / | + | 270 | Du et al, 2015 |
| Metabolic rate | | | | | | | | | | |
| *Margaritifera margaritifera* | *Salmo trutta* 0+ (H) | Ventilation control | 37 ± 8 gl/f | Glochidia load ¤ | 160 dpi | - | 0,012 | / | 50 | Thomas et al 2014 |
|  |  |  |  | Control * |  | - | <0.001 |  |  |  |
|  | *Salmo trutta* 1+ (W) | Standard Metabolic rate (SMR) | Between 0 and 11 gl/fg & ~66 gl/f | Glochidia load ¤ | ~250 dpi | - | 0,017 | −0.40 | 62 | Filipsson et al, 2017 |
|  |  | Max metabolic rate (MMR) |  |  |  | 0 | 0,81 | −0.08 |  |  |
|  |  | Standard Metabolic rate (SMR) |  | Control ¤ |  | + | 0,009 | / |  |  |
|  |  | Max metabolic rate (MMR) |  |  |  | + | 0,043 |  |  |  |
| *Sinanodonta woodiana* | *Rhodeus ocellatus* (W) | Initial MO2 | <120 gl/f on while body & ≤ 20 gl/f on gills | Control * | <1 dpi | 0 | 0,239 | / | 24 | Methling et al 2018 |
|  |  | Change in MO2 post stressor |  |  |  | - | 0,017 |  |  |  |
|  | *Cyprinus carpio* 4+ (H) | Electrocardiogram (EMG) | Infested at 4570 ± 1889 gl/l for 15 minutes in 15 liters | Control * | Average of 4 and 8 dpi | + | <0.001 | / | 22 | Slavik et al 2017 |
|  |  |  |  |  |  | + | <0.001 |  |  |  |
|  |  |  |  |  | 4 dpi | ~ + | / |  |  |  |
|  |  |  |  |  | 8 dpi | ~ 0 | / |  |  |  |
|  |  | Night EMG |  |  |  | - | 0,0001 |  |  |  |
|  |  | Day EMG |  |  |  | 0 | 0,0312 |  |  |  |
| *Hyriopsis cumingii* | *Pelteobagrus fulvidraco* | Rate of O consumption (MO2) | 2480 to 3244 gl/f | Control * | ~12 dpi | 0 | >0.05 | / | 270 | Du et al, 2015 |
|  |  | Ammonia excretion |  |  |  | + | <0.05 |  |  |  |
|  |  | Ammonia excretion |  | Glochidia load * | ~12 dpi | + | <0.05 | + |  |  |
| *Lampisilis reeveiana* | *Micropterus salmoides* 0+ | Ventilation quality | 632 gl/f | Glochidia Load ¤ | ~10 dpi | - | <0.001 | + | Unclear  58, 50, 35 | Kaiser, 2005 |
|  |  |  |  |  | ~90 dpi | - | <0.001 | + |  |  |
|  |  |  |  | Control * | ~10 dpi | - | <0.001 | / |  |  |
|  |  |  |  |  | ~90 dpi | - | <0.001 |  |  |  |
|  |  | Rate of O consumption (MO2) |  | Control * | avg of ~90 days | - | <0.05 | - |  |  |
|  |  | Tolerance to hypoxia (critical DO level) |  | Glochidia Load ¤ | 2 dpi | - |  | + |  |  |
|  |  |  |  |  | 9dpi | - | <0.001 |  |  |  |
|  |  |  |  |  | 14 dpi | - |  |  |  |  |
|  |  |  |  |  | 18dpi | - |  |  |  |  |
|  |  |  |  |  | 49 dpi | - |  |  |  |  |
|  |  |  |  |  | 63dpi | - | <0.05 |  |  |  |
|  |  |  |  |  | 89 dpi | - |  |  |  |  |
|  |  | Aquatic surface respiration |  | Control * | avg of ~90 days | - |  |  |  |  |
| *Lampsilis straminea* | *Lepomis macrochirus* | Tolerance to hypoxia (critical DO level) | 157.2 ± 35.8 gl/f | Control * | 1dpi | 0 | >0.05 | / | 88 | Horne, 2021 |
|  |  | Metabolic rate |  |  |  | 0 |  |  |  |  |
|  |  | Regulation Index |  |  |  | 0 |  |  |  |  |
|  |  | Tolerance to hypoxia (critical DO level) | 65.7 ± 36.5 |  | 2dpi | 0 |  |  |  |  |
|  |  | Metabolic rate |  |  |  | 0 |  |  |  |  |
|  |  | Regulation Index |  |  |  | 0 |  |  |  |  |
|  |  | Tolerance to hypoxia (critical DO level) | 40.8 ± 7.3 |  | 3dpi | 0 |  |  |  |  |
|  |  | Metabolic rate |  |  |  | 0 |  |  |  |  |
|  |  | Regulation Index |  |  |  | 0 |  |  |  |  |
|  |  | Tolerance to hypoxia (critical DO level) | 40.8 ± 7.3 |  | 7dpi | 0 |  |  |  |  |
|  |  | Metabolic rate |  |  |  | 0 |  |  |  |  |
|  |  | Regulation Index |  |  |  | 0 |  |  |  |  |
|  |  | Tolerance to hypoxia (critical DO level) | 41.5 ± 17.7 |  | 14dpi | 0 |  |  |  |  |
|  |  | Metabolic rate |  |  |  | 0 |  |  |  |  |
|  |  | Regulation Index |  |  |  | 0 |  |  |  |  |
|  |  | Tolerance to hypoxia (critical DO level) | 30.7 ± 16.9 |  | 21dpi | 0 |  |  |  |  |
|  |  | Metabolic rate |  |  |  | 0 |  |  |  |  |
|  |  | Regulation Index |  |  |  | 0 |  |  |  |  |
|  |  | Tolerance to hypoxia (critical DO level) | 11.5 ± 5.5 |  | 35dpi | 0 |  |  |  |  |
|  |  | Metabolic rate |  |  |  | 0 |  |  |  |  |
|  |  | Regulation Index |  |  |  | 0 |  |  |  |  |
|  |  | Tolerance to hypoxia (critical DO level) | 37.0 ± 16.9 |  | 56dpi | 0 |  |  |  |  |
|  |  | Metabolic rate |  |  |  | 0 |  |  |  |  |
|  |  | Regulation Index |  |  |  | 0 |  |  |  |  |
|  | *Micropterus salmoides* | Tolerance to hypoxia (critical DO level) | 151.8 ± 20.8 | Control* | 1dpi | 0 |  |  | 88 |  |
|  |  | Metabolic rate |  |  |  | 0 |  |  |  |  |
|  |  | Regulation Index |  |  |  | 0 |  |  |  |  |
|  |  | Tolerance to hypoxia (critical DO level) | 146.7 ± 11.1 |  | 2dpi | 0 |  |  |  |  |
|  |  | Metabolic rate |  |  |  | 0 |  |  |  |  |
|  |  | Regulation Index |  |  |  | 0 |  |  |  |  |
|  |  | Tolerance to hypoxia (critical DO level) | 138.3 ± 14.1 |  | 3dpi | 0 |  |  |  |  |
|  |  | Metabolic rate |  |  |  | 0 |  |  |  |  |
|  |  | Regulation Index |  |  |  | 0 |  |  |  |  |
|  |  | Tolerance to hypoxia (critical DO level) | 178.8 ± 38.9 |  | 7dpi | 0 |  |  |  |  |
|  |  | Metabolic rate |  |  |  | 0 |  |  |  |  |
|  |  | Regulation Index |  |  |  | 0 |  |  |  |  |
|  |  | Tolerance to hypoxia (critical DO level) | 133.8 ± 17.2 |  | 14dpi | 0 |  |  |  |  |
|  |  | Metabolic rate |  |  |  | 0 |  |  |  |  |
|  |  | Regulation Index |  |  |  | 0 |  |  |  |  |
|  |  | Tolerance to hypoxia (critical DO level) | 166.2 ± 11.0 |  | 21dpi | 0 |  |  |  |  |
|  |  | Metabolic rate |  |  |  | 0 |  |  |  |  |
|  |  | Regulation Index |  |  |  | 0 |  |  |  |  |
|  |  | Tolerance to hypoxia (critical DO level) | 138.0 ± 16.1 |  | 24dpi | 0 |  |  |  |  |
|  |  | Metabolic rate |  |  |  | 0 |  |  |  |  |
|  |  | Regulation Index |  |  |  | 0 |  |  |  |  |
|  |  | Tolerance to hypoxia (critical DO level) | 84.3 ± 18.4 |  | 35dpi | 0 |  |  |  |  |
|  |  | Metabolic rate |  |  |  | 0 |  |  |  |  |
|  |  | Regulation Index |  |  |  | 0 |  |  |  |  |
|  |  | Tolerance to hypoxia (critical DO level) | 95.5 ± 14.7 |  | 56dpi | 0 |  |  |  |  |
|  |  | Metabolic rate |  |  |  | 0 |  |  |  |  |
|  |  | Regulation Index |  |  |  | 0 |  |  |  |  |
|  |  | Tolerance to hypoxia (critical DO level) | 83.8 ± 8.8 |  | 77dpi | 0 |  |  |  |  |
|  |  | Metabolic rate |  |  |  | 0 |  |  |  |  |
|  |  | Regulation Index |  |  |  | 0 |  |  |  |  |
| *Venustaconcha pleasii* | *Etheostoma caeruleum* (W) | Ventilation quality | 73.5 ± 35.4 initially attached. 13.2 ± 11.8 juveniles recovered by 20 DPI | Control * | 2 dpi | - | 0,005 | / | 20 | Crane et al, 2011 |
|  |  |  |  |  | 4 dpi | 0 | >0.05 |  |  |  |
|  |  |  |  |  | 6 dpi | 0 |  |  |  |  |
|  |  |  |  |  | 8 dpi | 0 |  |  |  |  |
|  |  |  |  |  | 10 dpi | 0 |  |  |  |  |
|  |  |  |  |  | 12 dpi | 0 |  |  |  |  |
|  |  |  |  |  | 14 dpi | 0 |  |  |  |  |
|  |  |  |  |  | 16 dpi | 0 |  |  |  |  |
|  |  |  |  |  | 18 dpi | 0 |  |  |  |  |
|  |  |  |  |  | 20 dpi | 0 |  |  |  |  |
|  |  |  |  |  | 22 dpi | 0 |  |  |  |  |
| *Ptychobranchus occidentalis* |  | Ventilation quality | 31.5 ± 23.7 initially attached. 13.2 ± 16.7 juveniles recovered by 22 DPI | Control * | 2 dpi | 0 | > 0.05 | / | 36 |  |
|  |  |  |  |  | 8 dpi | 0 |  |  |  |  |
|  |  |  |  |  | 14 dpi | - | 0,009 |  |  |  |
|  |  |  |  |  | 20 dpi | 0 | > 0.05 |  |  |  |
|  |  |  |  |  | 28 dpi | 0 |  |  |  |  |
| *Anodonta anatina* | *Rhodeus amarus* (W) | Change in SMR | 19 gl/f on whole body & 2 gl/f on gills | Control * | 1 dpi | - | 0,009 | / | 48 | Methling et al 2019 |
|  |  |  |  |  | 2 dpi | 0 |  |  |  |  |
|  |  |  |  |  | 3 dpi | 0 |  |  |  |  |
|  |  |  |  |  | 4 dpi | + |  |  |  |  |
|  |  | SMR |  | Glochidia load ¤ | 4 dpi | - | 0,011 | + |  |  |
|  |  | Change in MO2 |  | Control * |  | 0 | 0,205 | / |  |  |
|  |  | MO2 Max |  | Glochidia Load ¤ |  | + | 0,048 | + |  |  |
| Organ Histology | | | | | | | | | | |
| *Margaritifera margaritifera* | *Salmo trutta* (H) | Spleen size | 100.7 (±18.62) gl/f (SD) | Control * | 15 dpi | 0 | > 0.05 | / | 27 | Thomas et al 2014 |
|  |  |  | 150.9 (±2.94) gl/f (SD) |  | 30 dpi | + | < 0.001 |  | 27 |  |
|  |  |  | 36.7 (±7.94) gl/f (SD) |  | 160 dpi | 0 | > 0.05 |  | 30 |  |
| *Sinanodonta woodiana* | *Squalius cephalus* (H) | Spleen size | 120.5 ± 39.3 gl/f (SD) | Control * | 43 dp first infestation, 3 dp last infestation | 0 | > 0.05 | / | 16 | Douda et al, 2017 |
|  |  |  | 24.6 ± 4.1 gl/f (SD) |  |  | 0 |  |  |  |  |
| *Strophitus undulatus* | *Oncorhynchus mykiss* (H) | Liver size | 26 ± 3 gl/f (SD) on whole body 8 ± 2 (SD) on gills | Control * | 28 dpi | 0 | > 0.05 | / | 9 | Defo et al, 2019 |
| Toxicology | | | | | | | | | | |
| *Anodonta oregone* | *Oncorhynchus kiutch* <1 month old (H) | 96h LC50 of naphthalene | 130 to 10 gl/f avg of 69 gl/f | Glochidia Load * | <1 dpi | - | / | / | 540 | Moles 1980 |
|  |  |  |  | Control * |  | - |  |  |  |  |
|  |  | 96-hour LC50 toluene | 110 to 5 gl/f avg of 69 gl/f | Glochidia Load * |  | - |  |  | 700 |  |
|  |  |  |  | Control * |  | - |  |  |  |  |
|  |  | 96-hour LC50 crude oil | 100 to 10 gl/f avg of 81 gl/f | Glochidia Load * |  | - |  |  | 420 |  |
|  |  |  |  | Control * |  | - |  |  |  |  |
| *Strophitus undulatus* | *Oncorhynchus mykiss* (H) | Liver cadmium concentration at baseline cadmium levels | 26 ± 3 gl/f (SD) on whole body 8 ± 2 (SD) on gills | Control * | 28 dpi | 0 | >0.05 | / | 77 | Defo et al, 2019 |
|  |  | Liver cadmium concentration with excess cadmium |  |  |  | 0 | >0.05 | / | 78 |  |
| Gene Transcription | | | | | | | | | | |
| *Strophitus undulatus* | *Oncorhynchus mykiss* (H) | Transcriptomic changes under glochidial stress | 26 ± 3 gl/f (SD) on whole body 8 ± 2 (SD) on gills | Control * | 28 dpi | 0 | / | / | 77 | Defo et al, 2019 |
|  |  | Transcriptomic changes to loc110530601 under combined glochidia and high cadmium stress in liver (immune system) |  | Not shared with high cadmium stress * |  | + |  |  | 78 |  |
|  |  | Transcriptomic changes to loc110514382 under combined glochidia and high cadmium stress in liver (immune system) |  |  |  | + |  |  |  |  |
|  |  | Transcriptomic changes to loc110504376 under combined glochidia and high cadmium stress in liver (malignant tumor) |  |  |  | - |  |  |  |  |
| *Elliptio complanata* | *Perca flavescens* 0+ (W) | Transcription of HSP70 in liver | 59 to 93 gl/f | Control * | 1 dpi | + | < 0.01 | / | 20 | Grendon, et al., 2019 |
|  |  | Transcription of g6pd in liver |  |  |  | 0 | >0.05 |  |  |  |
|  |  | Transcription of g6p in liver |  |  |  | 0 |  |  |  |  |
|  |  | Transcription of glyc in liver |  |  |  | - | < 0.0001 |  |  |  |
|  |  | Transcription of grlf1 in liver |  |  |  | - | < 0.001 |  |  |  |
|  |  | Transcription of HSP70 in gills |  |  |  | + | < 0.0001 |  |  |  |
|  |  | Transcription of g6pd in gills |  |  |  | 0 | >0.05 |  |  |  |
|  |  | Transcription of grlf1 in gills |  |  |  | 0 |  |  |  |  |
| *Lampsilis radiata* |  | Transcription of HSP70 in liver | 51 to 78 gl/f |  |  | + | < 0.01 |  |  |  |
|  |  | Transcription of g6pd in liver |  |  |  | 0 | >0.05 |  |  |  |
|  |  | Transcription of g6p in liver |  |  |  | 0 |  |  |  |  |
|  |  | Transcription of glyc in liver |  |  |  | 0 |  |  |  |  |
|  |  | Transcription of grlf1 in liver |  |  |  | 0 |  |  |  |  |
|  |  | Transcription of HSP70 in gills |  |  |  | 0 |  |  |  |  |
|  |  | Transcription of g6pd in gills |  |  |  | 0 |  |  |  |  |
|  |  | Transcription of grlf1 in gills |  |  |  | 0 |  |  |  |  |
| Coloration | | | | | | | | | | |
| *Margaritifera margaritifera* | *Salmo trutta* (W) | Dark coloration | 1 to 309 gl/f | Control ¤ | ~50 dpi | + | <0.001 | / | 14 | Filipsson et al, 2016 |
| *Anodonta anatina* | *Phoxinus phoxinus* (W) | Mating coloration | 5 ± 1 gl/f in high & 1 ± 0 gl/f in low | Glochidia Load * | 14 dpi | 0 | 0,48 | / | ~90 | Kekalainen et al, 2014 |
| Mating | | | | | | | | | | |
| *Anodonta anatina* | *Phoxinus phoxinus* (W) | Sperm motility including all individuals | 5 ± 1 gl/f in high & 1 ± 0 gl/f in low | Glochidia Load * | 14 dpi | 0 | 0,129 | / | ~90 | Kekalainen et al, 2014 |
|  |  | Sperm motility including only parasitized individuals |  |  |  | - | 0,011 |  |  |  |
|  |  | Curvature of sperm swimming trajectory |  |  |  | - | 0,039 |  |  |  |
|  |  | Breeding tubercle number |  |  |  | - | 0,047 |  |  |  |
| Molecular Changes (present in plasma unless otherwise specified) | | | | | | | | | | |
| *Sinanodonta woodiana* | *Cyprinus carpio* 4+ (H) | Hematocrit | Infested at 4570 ± 1889 gl/l for 15 minutes in 15 liters | Control * | 8 dpi | 0 | >0.01 | / | 22 | Slavik et al 2017 |
|  |  | Hemoglobin concentration |  |  |  | 0 | >0.01 |  |  |  |
|  |  | Cortisol |  |  |  | 0 | >0.01 |  |  |  |
|  |  | Aspartate aminotransferase |  |  |  | + | < 0.01 |  |  |  |
|  |  | Alanine aminotransferase |  |  |  | + | < 0.01 |  |  |  |
|  |  | Lactate dehydrogenase |  |  |  | 0 | >0.01 |  |  |  |
|  |  | Alaline phosphatase |  |  |  | 0 | >0.01 |  |  |  |
|  |  | Calcium (Ca) |  |  |  | 0 | >0.01 |  |  |  |
|  |  | Potassium (K) |  |  |  | + | < 0.01 |  |  |  |
|  |  | Sodium (Na) |  |  |  | 0 | >0.01 |  |  |  |
|  |  | Chloride (Cl) |  |  |  | + | < 0.01 |  |  |  |
|  | *Squalius cephalus* | Potassium (K) | Low load 24.6 ± 4.1 gl/f (SD) | Control * | 43 dp first infestation, 3 dp last infestation | + | <0.001 |  |  | Douda et al, 2017 |
|  |  | Sodium (Na) |  |  |  | 0 | >0.05 |  |  |  |
|  |  | Calcium (Ca) |  |  |  | 0 | >0.05 |  |  |  |
|  |  | Chloride (Cl) |  |  |  | + | <0.001 |  |  |  |
|  |  | Aspartate aminotransferase |  |  |  | 0 | >0.05 |  |  |  |
|  |  | Alanine aminotransferase |  |  |  | 0 | >0.05 |  |  |  |
|  |  | Lactate dehydrogenase |  |  |  | 0 | >0.05 |  |  |  |
|  |  | Alaline phosphatase |  |  |  | + | <0.05 |  |  |  |
|  |  | Cortisol |  |  |  | 0 | >0.05 |  |  |  |
|  |  | Potassium (K) | High load 120.5 ± 39.3 gl/f (SD) |  |  | + | <0.001 |  |  |  |
|  |  | Sodium (Na) |  |  |  | 0 | >0.05 |  |  |  |
|  |  | Calcium (Ca) |  |  |  | 0 | >0.05 |  |  |  |
|  |  | Chloride (Cl) |  |  |  | + | <0.001 |  |  |  |
|  |  | Aspartate aminotransferase |  |  |  | + | <0.01 |  |  |  |
|  |  | Alanine aminotransferase |  |  |  | + | <0.01 |  |  |  |
|  |  | Lactate dehydrogenase |  |  |  | + | <0.01 |  |  |  |
|  |  | Alaline phosphatase |  |  |  | + | <0.01 |  |  |  |
|  |  | Cortisol |  |  |  | 0 | >0.05 |  |  |  |
| *Margaritifera margaritifera* | *Salno trutta* (H) | Hematocrit | 150.9 (±2.94) gl/f (SD) | Control * | 30 dpi | 0 | 0,627 | / | 50 | Thomas et al 2014 |
|  |  |  |  | Glochidia Load ¤ |  | 0 | 0,17 |  | 21 |  |
|  | *Salmo trutta* 1+ (W) | Hematocrit | Between 0 and 11 gl/fg & ~66 gl/f | Control ¤ | ~250 dpi | + | 0,038 | / | 62 | Filipsson et al, 2017 |
|  | *Salmo trutta* 1+ (H) | Hematocrit | 212.8 gl/f | Control * | 300 dpi | 0 | 0,233 | / | 500 | Marwaha, et al 2019 |
|  |  |  |  | Glochidia Load ¤ |  | + | 0,029 | 0,33 |  |  |
|  | *Salmo salar* 1+ (H) | Chloride (Cl) | 66 to 219 gl/f (up to 1000 in some cases) | Control * | ~288 dpi | + | 0,0002 | / | ~40 | Treasurer & Turnbull 2000 |
| *Hyriopsis cumingii* | *Tilapia nilotica* | Total Protein | ~ > 24141 gl/f | Control * | ~ 7 dpi | 0 | >0.05 | / | ~ 8+ | Wen et al 2009 |
|  |  | Albumin |  |  |  | 0 |  |  |  |  |
|  |  | Glucose |  |  |  | 0 |  |  |  |  |
|  |  | Cholesterol |  |  |  | 0 |  |  |  |  |
|  |  | Triglyceride |  |  |  | 0 |  |  |  |  |
|  |  | Low Density Lipoprotein |  |  |  | 0 |  |  |  |  |
|  |  | High Density Lipoprotein |  |  |  | 0 |  |  |  |  |
|  |  | Total Protein | 0 gl/f | Control * | ~ 13 dpi | 0 | >0.05 | / | ~ 8+ |  |
|  |  | Albumin |  |  |  | 0 |  |  |  |  |
|  |  | Glucose |  |  |  | 0 |  |  |  |  |
|  |  | Cholesterol |  |  |  | 0 |  |  |  |  |
|  |  | Triglyceride |  |  |  | - | 0,001 |  |  |  |
|  |  | Low Density Lipoprotein |  |  |  | - | 0,02 |  |  |  |
|  |  | High Density Lipoprotein |  |  |  | 0 | >0.05 |  |  |  |
|  | *Pelteobagrus fulvidraco* (H) | Liver Glycogen | / | Control * | 2 dpi | 0 | > 0.05 | / | 120 | Ma et al 2018 |
|  |  | Liver Free Fatty Acid |  |  |  | 0 |  |  |  |  |
|  |  | Liver Total Protein |  |  |  | 0 |  |  |  |  |
|  |  | Liver Total Amino Acid |  |  |  | 0 |  |  |  |  |
|  |  | Muscle Glycogen |  |  |  | 0 |  |  |  |  |
|  |  | Muscle Free fatty Acid |  |  |  | 0 |  |  |  |  |
|  |  | Muscle Total Protein |  |  |  | 0 |  |  |  |  |
|  |  | Muscle Total Amino Acid |  |  |  | 0 |  |  |  |  |
|  |  | Plasma Free Fatty Acid |  |  |  | 0 |  |  |  |  |
|  |  | Plasma Total Protein |  |  |  | 0 |  |  |  |  |
|  |  | Plasma Total Amino Acid |  |  |  | 0 |  |  |  |  |
|  |  | Liver Glycogen |  |  | 5 dpi | 0 | > 0.05 |  |  |  |
|  |  | Liver Free Fatty Acid |  |  |  | 0 |  |  |  |  |
|  |  | Liver Total Protein |  |  |  | 0 |  |  |  |  |
|  |  | Liver Total Amino Acid |  |  |  | 0 |  |  |  |  |
|  |  | Muscle Glycogen |  |  |  | 0 |  |  |  |  |
|  |  | Muscle Free fatty Acid |  |  |  | 0 |  |  |  |  |
|  |  | Muscle Total Protein |  |  |  | 0 |  |  |  |  |
|  |  | Muscle Total Amino Acid |  |  |  | 0 |  |  |  |  |
|  |  | Plasma Free Fatty Acid |  |  |  | 0 |  |  |  |  |
|  |  | Plasma Total Protein |  |  |  | 0 |  |  |  |  |
|  |  | Plasma Total Amino Acid |  |  |  | - | <0.05 |  |  |  |
|  |  | Liver Glycogen |  |  | 12 dpi | 0 | > 0.05 |  |  |  |
|  |  | Liver Free Fatty Acid |  |  |  | - | <0.05 |  |  |  |
|  |  | Liver Total Protein |  |  |  | 0 | > 0.05 |  |  |  |
|  |  | Liver Total Amino Acid |  |  |  | - | <0.05 |  |  |  |
|  |  | Muscle Glycogen |  |  |  | 0 | > 0.05 |  |  |  |
|  |  | Muscle Free fatty Acid |  |  |  | 0 |  |  |  |  |
|  |  | Muscle Total Protein |  |  |  | 0 |  |  |  |  |
|  |  | Muscle Total Amino Acid |  |  |  | 0 |  |  |  |  |
|  |  | Plasma Free Fatty Acid |  |  |  | 0 |  |  |  |  |
|  |  | Plasma Total Protein |  |  |  | 0 |  |  |  |  |
|  |  | Plasma Total Amino Acid |  |  |  | - | <0.05 |  |  |  |
| *Hyriopsis myersiana* | Average of *Cyprinus carprio* & *Oreochromis nilotica* & *Clarias macrocephalus x C. gariepinus* & *Pangasius pangasius* | Total Protein | Infested with 2662.5 to 129.4 glochidia per  branchial chamber, the chambers being kept closed for  2 min | Control * | 3 dpi | + | <0.01 | / | 48 | Uthaiwan, et al, 2003 |
|  |  | Triglyceride |  |  |  | 0 | >0.05 |  |  |  |
|  |  | Glucose |  |  |  | 0 |  |  |  |  |
|  |  | Copper (Cu) |  |  |  | 0 |  |  |  |  |
|  |  | Manganese (Mn) |  |  |  | 0 |  |  |  |  |
|  |  | Phosphorus (P) |  |  |  | 0 |  |  |  |  |
|  |  | Sulfur (S) |  |  |  | 0 |  |  |  |  |
|  |  | Sodium (Na) |  |  |  | 0 |  |  |  |  |
|  |  | Calcium (Ca) |  |  |  | 0 |  |  |  |  |
|  |  | Potassium (K) |  |  |  | - | <0.05 |  |  |  |
|  |  | Magnesium (Mg) |  |  |  | 0 | >0.05 |  |  |  |
|  |  | Chloride (Cl) |  |  |  | 0 |  |  |  |  |
|  |  | Osmolality |  |  |  | 0 |  |  |  |  |
|  |  | Total Protein |  |  | 6 dpi | 0 | >0.05 |  |  |  |
|  |  | Triglyceride |  |  |  | 0 |  |  |  |  |
|  |  | Glucose |  |  |  | 0 |  |  |  |  |
|  |  | Copper (Cu) |  |  |  | 0 |  |  |  |  |
|  |  | Manganese (Mn) |  |  |  | 0 |  |  |  |  |
|  |  | Phosphorus (P) |  |  |  | 0 |  |  |  |  |
|  |  | Sulfur (S) |  |  |  | 0 |  |  |  |  |
|  |  | Sodium (Na) |  |  |  | 0 |  |  |  |  |
|  |  | Calcium (Ca) |  |  |  | 0 |  |  |  |  |
|  |  | Potassium (K) |  |  |  | 0 |  |  |  |  |
|  |  | Magnesium (Mg) |  |  |  | - | <0.01 |  |  |  |
|  |  | Chloride (Cl) |  |  |  | 0 | >0.05 |  |  |  |
|  |  | Osmolality |  |  |  | 0 |  |  |  |  |
|  |  | Total Protein |  |  | 12 dpi | + | <0.01 |  |  |  |
|  |  | Triglyceride |  |  |  | + | <0.01 |  |  |  |
|  |  | Glucose |  |  |  | 0 | >0.05 |  |  |  |
|  |  | Copper (Cu) |  |  |  | 0 |  |  |  |  |
|  |  | Manganese (Mn) |  |  |  | 0 |  |  |  |  |
|  |  | Phosphorus (P) |  |  |  | 0 |  |  |  |  |
|  |  | Sulfur (S) |  |  |  | 0 |  |  |  |  |
|  |  | Sodium (Na) |  |  |  | 0 |  |  |  |  |
|  |  | Calcium (Ca) |  |  |  | + | 0,05 |  |  |  |
|  |  | Potassium (K) |  |  |  | 0 | >0.05 |  |  |  |
|  |  | Magnesium (Mg) |  |  |  | - | <0.01 |  |  |  |
|  |  | Chloride (Cl) |  |  |  | - | <0.05 |  |  |  |
|  |  | Osmolality |  |  |  | 0 | >0.05 |  |  |  |
|  | *Cyprinus carprio* | Total Protein |  |  | 12 dpi | 0 | >0.05 | / | 6 |  |
|  |  | Glucose |  |  |  | 0 |  |  |  |  |
|  |  | Triglyceride |  |  |  | 0 |  |  |  |  |
|  |  | Copper (Cu) |  |  |  | 0 |  |  |  |  |
|  |  | Phosphorus (P) |  |  |  | 0 |  |  |  |  |
|  |  | Manganese (Mn) |  |  |  | 0 |  |  |  |  |
|  |  | Magnesium (Mg) |  |  |  | 0 |  |  |  |  |
|  |  | Calcium (Ca) |  |  |  | 0 |  |  |  |  |
|  |  | Sulfur (S) |  |  |  | 0 |  |  |  |  |
|  |  | Sodium (Na) |  |  |  | 0 |  |  |  |  |
|  |  | Chloride (Cl) |  |  |  | 0 |  |  |  |  |
|  |  | Osmolality |  |  |  | 0 |  |  |  |  |
|  | *Oreochromis nilotica* | Total Protein |  |  |  | 0 | >0.05 |  | 6 |  |
|  |  | Glucose |  |  |  | 0 |  |  |  |  |
|  |  | Triglyceride |  |  |  | - | <0.01 |  |  |  |
|  |  | Copper (Cu) |  |  |  | 0 | >0.05 |  |  |  |
|  |  | Phosphorus (P) |  |  |  | 0 |  |  |  |  |
|  |  | Manganese (Mn) |  |  |  | 0 |  |  |  |  |
|  |  | Magnesium (Mg) |  |  |  | + | 0,05 |  |  |  |
|  |  | Calcium (Ca) |  |  |  | 0 | >0.05 |  |  |  |
|  |  | Sulfur (S) |  |  |  | 0 |  |  |  |  |
|  |  | Sodium (Na) |  |  |  | 0 |  |  |  |  |
|  |  | Chloride (Cl) |  |  |  | 0 |  |  |  |  |
|  |  | Osmolality |  |  |  | 0 |  |  |  |  |
|  | *Clarias macrocephalus x C. gariepinus* | Total Protein |  | Control ¤ |  | 0 | >0.05 |  | 6 |  |
|  |  | Glucose |  |  |  | 0 |  |  |  |  |
|  |  | Triglyceride |  |  |  | - | <0.01 |  |  |  |
|  |  | Copper (Cu) |  |  |  | 0 | >0.05 |  |  |  |
|  |  | Phosphorus (P) |  |  |  | 0 |  |  |  |  |
|  |  | Manganese (Mn) |  |  |  | 0 |  |  |  |  |
|  |  | Calcium (Ca) |  |  |  | 0 |  |  |  |  |
|  |  | Magnesium (Mg) |  |  |  | 0 |  |  |  |  |
|  |  | Potassium (K) |  |  |  | 0 |  |  |  |  |
|  |  | Sulfur (S) |  |  |  | + | <0.05 |  |  |  |
|  |  | Sodium (Na) |  |  |  | 0 | >0.05 |  |  |  |
|  |  | Chloride (Cl) |  |  |  | 0 |  |  |  |  |
|  |  | Osmolality |  |  |  | 0 |  |  |  |  |
|  | *Pangasius pangasius* | Total Protein |  |  |  | 0 | >0.05 |  | 6 |  |
|  |  | Glucose |  |  |  | 0 |  |  |  |  |
|  |  | Triglyceride |  |  |  | - | <0.05 |  |  |  |
|  |  | Copper (Cu) |  |  |  | 0 | >0.05 |  |  |  |
|  |  | Phosphorus (P) |  |  |  | 0 |  |  |  |  |
|  |  | Manganese (Mn) |  |  |  | 0 |  |  |  |  |
|  |  | Calcium (Ca) |  |  |  | 0 |  |  |  |  |
|  |  | Magnesium (Mg) |  |  |  | 0 |  |  |  |  |
|  |  | Potassium (K) |  |  |  | 0 |  |  |  |  |
|  |  | Sulfur (S) |  |  |  | + | <0.01 |  |  |  |
|  |  | Sodium (Na) |  |  |  | 0 | >0.05 |  |  |  |
|  |  | Chloride (Cl) |  |  |  | + | <0.01 |  |  |  |
|  |  | Osmolality |  |  |  | 0 | >0.05 |  |  |  |
| *Utterbackia imbecillis* | *Lepomis macrochirus* | Hematocrit | 25 minutes in 1000 gl/l in 19l | Control * | 1 dpi | - | < 0.05 | / | 24 | Dubansky et al 2011 |
|  |  | Sodium (Na) |  |  |  | 0 | > 0.05 |  |  |  |
|  |  | Glucose |  |  |  | 0 |  |  |  |  |
|  |  | Lactate |  |  |  | 0 |  |  |  |  |
|  |  | Cortisol |  |  |  | 0 |  |  |  |  |
|  |  | Hematocrit | 25 minutes in 2000 gl/l in 19l |  |  | 0 | > 0.05 |  |  |  |
|  |  | Sodium (Na) |  |  |  | 0 |  |  |  |  |
|  |  | Glucose |  |  |  | 0 |  |  |  |  |
|  |  | Lactate |  |  |  | 0 |  |  |  |  |
|  |  | Cortisol |  |  |  | + | < 0.05 |  |  |  |
|  |  | Hematocrit | 25 minutes in 4000 gl/l in 19l |  |  | - | < 0.05 |  |  |  |
|  |  | Sodium (Na) |  |  |  | 0 | > 0.05 |  |  |  |
|  |  | Glucose |  |  |  | 0 |  |  |  |  |
|  |  | Lactate |  |  |  | 0 |  |  |  |  |
|  |  | Cortisol |  |  |  | + | < 0.05 |  |  |  |
|  |  | Hematocrit | 25 minutes in 8000 gl/l in 19l |  |  | 0 | > 0.05 |  |  |  |
|  |  | Sodium (Na) |  |  |  | 0 |  |  |  |  |
|  |  | Glucose |  |  |  | 0 |  |  |  |  |
|  |  | Lactate |  |  |  | 0 |  |  |  |  |
|  |  | Cortisol |  |  |  | + | < 0.05 |  |  |  |
