# Supplementary material for: Effects of parasitic freshwater mussels on their host fishes: a review
Source: Parasitology. 2022 Sep 2;149(14):1958–75. doi: 10.1017/S0031182022001226 (PMC10090606; doi:10.1017/S0031182022001226)
Supplement: Supplementary file 1 [file S0031182022001226sup.zip › S0031182022001226sup003.docx]

**Supplementary Table S3**

| Legend | |
| --- | --- |
| * | Manipulative study |
| ¤ | Non-manipulative study |
| ~ | Value not specified in paper, circumstantial information provided in paper informs an estimated value. Accuracy of estimations vary. |
| gl/f | glochidia per fish |
| gl/fg | glochidia per gram of fish |
| dpi | days post infestation |
| (W) | Wild caught fish |
| (H) | Hatchery reared fish |

| Mussel species | Fish species | Specific behavior | Glochidia load | Against | DPI | +/-/0 | p | Correlation Coefficient | n= | Paper |
| --- | --- | --- | --- | --- | --- | --- | --- | --- | --- | --- |
| *Margaitifera margaritifera* | *Salmo trutta* (H) | Swimming performance | High infestation level: 906 ± 156 gl/fg | Control * | 1 dpi | 0 | >0.05 | / | 12 | Taeubert & Geist 2013 |
|  |  | Swimming performance / body length |  |  |  | 0 |  |  |  |  |
|  |  | Swimming performance | High medium infestation level: 353 ± 91 gl/fg |  |  | 0 |  |  | 16 |  |
|  |  | Swimming performance / body length |  |  |  | 0 |  |  |  |  |
|  |  | Swimming performance | Low medium infestation level: 113 ± 20 gl/fg |  |  | 0 |  |  |  |  |
|  |  | Swimming performance / body length |  |  |  | 0 |  |  |  |  |
|  |  | Swimming performance | Low infestation level: 113 ± 20 gl/fg |  |  | 0 |  |  |  |  |
|  |  | Swimming performance / body length |  |  |  | 0 |  |  |  |  |
|  |  | Swimming performance | Between 0gl/fg and 906 ± 156 gl/fg | Glochidial load * |  | - | 0,004 | -0,469 | 36 |  |
|  |  | Swimming performance / body length |  |  |  | - | 0,002 | -0,5 |  |  |
|  |  | Feeding rate | 25.1±5.7 gl/fg | Control * | ~57dpi | - | 0,017 | / | 10 | Österling et al, 2014 |
|  |  | Starvation feeding rate |  |  |  | - | <0.05 |  |  |  |
|  |  | satiated feeding rate |  |  |  | - | <0.05 |  |  |  |
|  |  | Close feeding |  |  |  | 0 | >0.05 |  |  |  |
|  |  | Distant feeding |  |  |  | - | <0.05 |  |  |  |
|  |  | Average distant feeding |  |  |  | - | 0,025 |  |  |  |
|  |  | Foraging | 326.7 ± 187.9 gl/f | Control * | 14 dpi | 0 | >0.05 | / | 60 | Höglund, 2014 |
|  |  |  |  |  | 28 dpi | 0 |  |  |  |  |
|  |  |  |  |  | 42 dpi | 0 |  |  |  |  |
|  |  |  |  |  | 56 dpi | 0 |  |  |  |  |
|  |  |  |  |  | 70 dpi | 0 |  |  |  |  |
|  |  |  |  |  | Over 70 days | 0 |  |  |  |  |
|  |  | Foraging efficiency (spitting food) |  |  |  | - | 0,01 |  |  |  |
|  |  | Activity levels |  |  | 14 dpi | 0 | >0.05 |  |  |  |
|  |  |  |  |  | 28 dpi | 0 |  |  |  |  |
|  |  |  |  |  | 42 dpi | 0 |  |  |  |  |
|  |  |  |  |  | 56 dpi | 0 |  |  |  |  |
|  |  |  |  |  | 70 dpi | 0 |  |  |  |  |
|  |  |  |  |  | Over all 70 days | 0 |  |  |  |  |
|  |  | Foraging alone | Low infection 0.71 ± 0.59 gl/f High infection 202 ± 58 gl/f | Control * | ~8 dpi | 0 | >0.05 | / | 40 | Sunnerstam, 2013 |
|  |  | Foraging in competition |  |  |  | 0 |  |  |  |  |
|  |  | Foraging alone |  | Low infestation ¤ | ~62 dpi | - | 0,013 |  | 12 |  |
|  |  | Foraging in competition |  |  |  | - | 0,035 |  |  |  |
|  | *Salmo trutta* (W) | Body Temperature | Infested with 8527±2970 gl/l for 15 mins | Control * | Observed over ~330 | - | <0.0161 | / | 49 | Horkey et al, 2019 |
|  |  | Body Temperature site 1 |  |  | ~87 dpi | 0 | >0.05 |  |  |  |
|  |  |  |  |  | ~177 dpi | 0 | >0.05 |  |  |  |
|  |  |  |  |  | ~269 dpi | - | 0,0486 |  |  |  |
|  |  |  |  |  | ~330 dpi | 0 | >0.05 |  |  |  |
|  |  | Body temperature site 2 |  |  | ~87 dpi | 0 | >0.05 |  |  |  |
|  |  |  |  |  | ~177 dpi | + | 0,0135 |  |  |  |
|  |  |  |  |  | ~269 dpi | 0 | >0.05 |  |  |  |
|  |  |  |  |  | ~330 dpi | 0 | >0.05 |  |  |  |
|  |  | Body temperature site 3 |  |  | ~177 dpi | 0 | >0.05 |  |  |  |
|  |  | Movement (relocation) |  |  | Observed over ~330 | - | <0.0001 |  |  |  |
|  |  | Movement (longitudinally) |  |  |  | + | <0.0251 |  |  |  |
|  |  | Movement (distance) |  |  |  | 0 | >0.05 |  |  |  |
|  |  | Home range size |  |  |  | - | <0.0167 |  |  |  |
|  |  | Diel movement |  |  | ~87 dpi | 0 | >0.05 |  |  |  |
|  |  |  |  |  | ~177 dpi | 0 |  |  |  |  |
|  |  |  |  |  | ~269 dpi | 0 |  |  |  |  |
|  |  |  |  |  | ~330 dpi | + | <0.0001 |  |  |  |
|  |  |  |  |  | Observed over ~330 | + | <0.0205 |  |  |  |
|  |  | Day diel movement |  |  | Observed over ~330 | ~ 0 | / |  |  |  |
|  |  | Twilight diel movement |  |  |  | ~ + |  |  |  |  |
|  |  | Night diel movement |  |  |  | ~ + |  |  |  |  |
|  |  | Foraging | 1 to 309 gl/f | Control ¤ | ~50 dpi | 0 | >0.05 | / | 28 | Filipsson et al, 2016 |
|  |  | Activity Level |  |  |  | 0 |  |  |  |  |
|  |  | Interactions initiated |  |  |  | 0 |  |  |  |  |
|  |  | Foraging |  | Glochidia load ¤ |  | - | 0,02 | -0,61 | 14 |  |
|  |  | Activity Level |  |  |  | - | 0,005 | -0,7 |  |  |
|  |  | Interactions initiated |  |  |  | - | 0,004 | -0,72 |  |  |
|  |  | Prey consumed homogenous habitat competition | High infestation > 100 gl/f Low infestation <30gl/f | Low Infestation ¤ | ~30 dpi | 0 | / | / | 28 | Gustavsson 2019 |
|  |  | Prey consumed complex habitat competition |  |  |  | 0 |  |  |  |  |
|  |  | Activity Level homogenous habitat competition |  |  |  | 0 |  |  |  |  |
|  |  | Activity Level complex habiat competition |  |  |  | 0 |  |  |  |  |
|  |  | Interactions initiated homogenous habitat competition |  |  |  | 0 |  |  |  |  |
|  |  | Interactions initiated complex habitat competition |  |  |  | + |  |  |  |  |
|  |  | Water velocity | / | Control * | 65 - 75 dpi | 0 | 0,06 | / | 62 | Andersson, 2018 |
|  |  | Water depth |  |  |  | 0 | 0,32 |  |  |  |
|  |  | Substrate size |  |  |  | - | 0,02 |  |  |  |
|  |  | Distance Moved | High infestation > 226 ± 24 Low infestation <18±15 | Low Infestation ¤ | ~100 dpi | + | 0,03 | / | 33 | Freitt, 2016 |
|  |  | Water depth |  |  |  | 0 | >0.05 |  |  |  |
|  |  | Water velocity |  |  |  | 0 |  |  |  |  |
|  |  | Substrate size |  |  |  | 0 |  |  |  |  |
|  |  | Distance from shore |  |  |  | 0 |  |  |  |  |
| *Ptychobranchus occidentalis* | *Etheostoma caeruleum* (W) | Moves to capture food | 73.5 ± 35.4 initially attached. 13.2 ± 11.8 juveniles recovered by 20 DPI | Control * | 2 dpi | 0 | > 0.05 | / | 20 | Crane et al, 2011 |
|  |  |  |  |  | 8 dpi | - | 0,03 |  |  |  |
|  |  |  |  |  | 14 dpi | 0 | > 0.05 |  |  |  |
|  |  |  |  |  | 20 dpi | - | 0,017 |  |  |  |
|  |  |  |  |  | 28 dpi | - | 0,033 |  |  |  |
|  |  | Prey consumed |  |  | 2 dpi | 0 | > 0.05 |  |  |  |
|  |  |  |  |  | 8 dpi | 0 |  |  |  |  |
|  |  |  |  |  | 14 dpi | 0 |  |  |  |  |
|  |  |  |  |  | 20 dpi | 0 |  |  |  |  |
|  |  |  |  |  | 28 dpi | 0 |  |  |  |  |
| *Venustaconcha pleasii* |  | Moves to capture food | 31.5 ± 23.7 initially attached. 13.2 ± 16.7 juveniles recovered by 22 DPI | Control * | 14 dpi | ~0 | / | / | 44 |  |
|  |  | Moves to capture food under predation stress |  |  |  | ~ + |  |  |  |  |
|  |  | Prey consumed |  |  | 14 dpi | ~ 0 | / |  |  |  |
|  |  | Prey consumed under predation stress |  |  |  | ~ + |  |  |  |  |
| *Anodonta anatina* | *Squalius cephalus* | Activity Level | trial 1: 67.8 ± 20.5 gl/fg trial 2: 48.9 ± 5.9 gl/fg | Control * | ~4 dpi | - | 0,0331 | / | 140 | Horky et al, 2014 |
|  |  |  |  |  | ~7 dpi | - | 0,0331 |  |  |  |
|  |  |  |  |  | ~12 dpi | 0 | >0.05 |  |  |  |
|  |  | Migration levels | trial 1: 75.6 ± 37.7 gl/fg trial 2: 66.5 ± 42.0 gl/fg |  | Observed over 30 days | - | <0.0261 |  | 743 |  |
|  |  | Temperature of migration |  |  |  | + | < 0.0013 |  |  |  |
|  |  | Distance from river bank | 32.9 ± 12.6 gl/fg |  | Observed over 30 days | + | < 0.0098 |  | 40 |  |
|  |  | Rate of movement |  |  |  | 0 | >0.05 |  |  |  |
|  |  | Timing of activity |  |  |  | 0 |  |  |  |  |
|  |  | Reaction to environmental variation |  |  |  | 0 |  |  |  |  |
| 38 potential assorted species | 4 Families: Centrarchidae, Ictaluridae, Sciaenidae, Catostomidae | Upstream migration | / | Observation ¤ | ~ 56 dpi | + | <0.001 | / | 89 | Irmscher & Vaughn 2015 |
| *Margaritifera laevis* | *Oncorhynchus masou masou* | Upstream migration | 48+29 gl/f | Control * | ~50 dpi | 0 | / | / | 215 | Terui et al, 2017 |
|  |  | Small fish upstream migration |  |  |  | - | / |  |  |  |
|  |  | Big fish Upstream migration |  |  |  | + | / |  |  |  |
| *Sinanodonta woodiana* | *Cyprinus carpio* 4+ (H) | Movement | Infested at 4570 ± 1889 gl/l for 15 minutes in 15 liters | Control * | 4 dpi | - | <0.0312 | / | 22 | Slavik et al, 2017 |
|  |  |  |  |  | 8 dpi | 0 | >0.05 |  |  |  |
|  |  | Night movement |  |  |  | 0 |  |  |  |  |
|  |  | Day movement |  |  |  | 0 |  |  |  |  |
